# Real‐Time Ferroelectric Domain Wall Dynamics During Electric Poling and Depoling

**DOI:** 10.1002/advs.76513

**Published:** 2026-07-09

**Authors:** Ziqi Wang, Zhengze Xu, Anastasia Timofeeva, Hossam Elnaggar, Sipan Liu, Yusen Pei, Reece Henry, Brendan O'Connor, Eunkyoung Shim, Franky So, Kara Peters, Xiaoning Jiang, Jun Liu

**Affiliations:** ^1^ Department of Mechanical and Aerospace Engineering North Carolina State University Raleigh North Carolina USA; ^2^ Department of Materials Science and Engineering North Carolina State University Raleigh North Carolina USA; ^3^ Department of Textile Engineering North Carolina State University Raleigh North Carolina USA

**Keywords:** domain wall dynamics, electrical depoling, electrical poling, ferroelectrics, relaxor‐PT crystals

## Abstract

Electrical poling protocols, including alternating current poling (ACP), direct current poling (DCP), and electrical depoling (EDP), are widely used to optimize the electromechanical properties of relaxor‐lead titanate (PT) ferroelectric single crystals. However, the microscopic mechanisms governing their distinct outcomes remain unresolved, largely due to the lack of direct, real‐time, and in‐situ experimental access to domain wall dynamics during poling. As a result, competing interpretations based on domain refinement, domain coarsening, or polarization switching have emerged from ex‐situ imaging and bulk‐averaged electromechanical measurements. Here, we track domain wall‐related birefringence dynamics in [110]‐oriented lead indium niobate‐lead magnesium niobate‐lead titanate single crystals during ACP, DCP, and EDP using instant polarized light microscopy π (IPOLπ). This single‐shot, non‐destructive technique enables continuous, real‐time tracking of domain wall nucleation, motion, and reconfiguration throughout the poling/depoling process. We reveal distinct, field‐dependent dynamic pathways for different electrical protocols, demonstrating pronounced path dependence and reversibility that are not evident from static domain configurations alone. These results identify domain wall dynamics as the dominant mechanism governing electrical poling and depoling in relaxor‐PT ferroelectrics and provide a dynamic framework for rational domain wall engineering in high‐performance electromechanical materials.

## Graphical abstract

Real‐time visualization of domain wall dynamics during alternating‐current poling, direct‐current poling, and electrical depoling in relaxor‐PT ferroelectric single crystals reveals distinct field‐dependent pathways and strong path dependence. Instant polarized light microscopy uncovers reversible domain wall nucleation, motion, and reconfiguration, establishing domain wall dynamics as the governing mechanism of electromechanical property evolution.

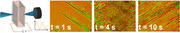

## 1 Introduction

Ferroelectric materials underpin a wide range of advanced electromechanical device technologies, including non‐volatile memories, nanoelectronic components, and precision sensors, owing to their switchable spontaneous polarization and strong electromechanical coupling [1, 2, 3, 4]. In these materials, polarization is organized into domains, regions of uniformly aligned dipoles, separated by domain walls, whose structure and mobility critically influence macroscopic dielectric, piezoelectric, and electromechanical responses [2, 5, 6, 7]. Beyond serving as passive boundaries, domain walls can act as active functional entities, and the controlled manipulation of domain configurations, often referred to as domain wall engineering, has emerged as a powerful strategy for optimizing ferroelectric performance [2, 5, 8].

Among ferroelectrics, relaxor‐based single crystals such as lead magnesium niobate‐lead titanate (PMN–PT), lead indium niobate‐lead magnesium niobate‐lead titanate (PIN–PMN–PT), and lead zinc niobate‐lead titanate (PZN–PT) exhibit exceptionally high piezoelectric coefficients, large dielectric permittivity, and pronounced field‐ and frequency‐dependent tunability, making them central to high‐performance actuator and transducer applications [9, 10, 11, 12, 13]. Electrical poling is the most widely adopted method for domain wall engineering in these materials, with direct current poling (DCP) and alternating current poling (ACP) being the two most commonly used approaches [14, 15, 16, 17, 18, 19, 20, 21]. More recently, electrical depoling (EDP) has been introduced as a reversible, field‐driven route to depolarize relaxor‐PT crystals without thermal treatment [22]. Despite their widespread use, these electrical poling protocols produce markedly different electromechanical outcomes, and the associated microscopic origins remain debated [14, 15, 16, 23, 24].

A growing body of work reports that ACP can yield superior electromechanical properties and reduce fatigue compared with DCP [14, 20, 21, 25, 26, 27, 28]. However, the physical mechanisms responsible for these improvements are far from settled. Competing interpretations have attributed enhanced properties under ACP to fundamentally different domain evolution scenarios, including domain refinement and increased domain‐wall density, domain coarsening and improved strain accommodation [5, 6, 7, 29], or enhanced polarization rotation within large domains [30, 31, 32, 33]. Critically, bulk‐averaged electromechanical metrics and static post‐mortem imaging cannot unambiguously distinguish between domain‐wall motion and polarization switching, as both mechanisms can produce similar macroscopic signatures [24, 34, 35, 36, 37, 38]. Consequently, it remains unclear whether domain evolution during electrical poling follows a deterministic pathway toward the final state or is instead strongly history‐dependent, governed by the initial domain configuration, field waveform, and cycling protocol [23, 24, 39].

This long‐standing ambiguity stems from a fundamental experimental limitation: the lack of direct, real‐time access to domain‐wall dynamics during electrical poling. Most prior studies rely on comparisons between pre‐ and post‐poling states using techniques such as piezoresponse force microscopy (PFM), X‐ray diffraction (XRD), and polarized light microscopy (PLM) [8, 14, 40]. While powerful, these approaches either probe only near‐surface regions or provide spatially averaged information, precluding direct visualization of domain‐wall nucleation, motion, interaction, and annihilation as they occur. Optical second‐harmonic generation (SHG) also provides a powerful non‐invasive optical probe of ferroic order and domain configurations, although its symmetry‐dependent nonlinear optical contrast and signal‐integration requirements make continuous real‐time, in‐situ imaging of complex electric‐field‐driven domain‐wall dynamics less straightforward [41, 42, 43]. In‐situ methods, including diffraction‐based techniques, transmission electron microscopy, and scanning probe approaches, have provided valuable insights into field‐induced structural evolution and switching behavior, yet they typically require nanoscale specimens or sacrifice spatial or temporal coverage, limiting their applicability to bulk single crystals under realistic poling conditions [24, 44, 45, 46, 47]. As a result, the dynamic processes that govern electrical poling in relaxor ferroelectrics remain largely inferred rather than directly observed.

Optical birefringence‐based imaging offers a non‐destructive pathway to overcome these challenges, as birefringence is intrinsically sensitive to changes in crystallographic orientation and strain fields associated with domain walls [48, 49]. Conventional polarized light microscopy, however, relies on sequential acquisition of multiple polarization states, restricting its use primarily to static or quasi‐static observations [8, 48]. Recent advances using dispersive quartz plates enable wavelength‐dependent polarization rotation, allowing polarization information to be spectrally encoded and retrieved within a single exposure [50, 51]. This principle underpins instant polarized light microscopy π (IPOLπ), which achieves single‐shot polarization‐resolved imaging with high temporal resolution [51, 52, 53]. By directly mapping spatial variations in optical axis orientation, IPOLπ is uniquely suited for capturing domain‐wall‐mediated responses in real time [54].

Here, we employ IPOLπ to directly visualize domain‐wall dynamics related optical changes from captured images in [110]‐oriented PIN–PMN–PT single crystals during ACP, DCP, and EDP processes. We chose the [110] orientation because it provides a domain‐wall geometry with strong optical contrast and well‐defined wall traces, enabling quantitative in‐situ tracking of domain‐wall dynamics with IPOLπ under ACP/DCP/EDP conditions. This single‐shot, non‐destructive approach enables continuous in‐situ tracking of domain‐wall nucleation, motion, and reconfiguration under different electrical field waveforms. By correlating time‐resolved optical polarization responses with applied electric fields, we identify distinct domain‐wall dynamic behaviors that underpin the path dependence and reversibility of electrical poling. These results provide direct experimental access to the internal dynamics of electrical poling and establish domain‐wall dynamics, rather than static domain configurations, as the central determinant of electromechanical properties in relaxor‐PT single crystals.

## 2 Results and Discussion

### 2.1 In Situ Visualization of Domain Dynamics During ACP

**FIGURE 1:**
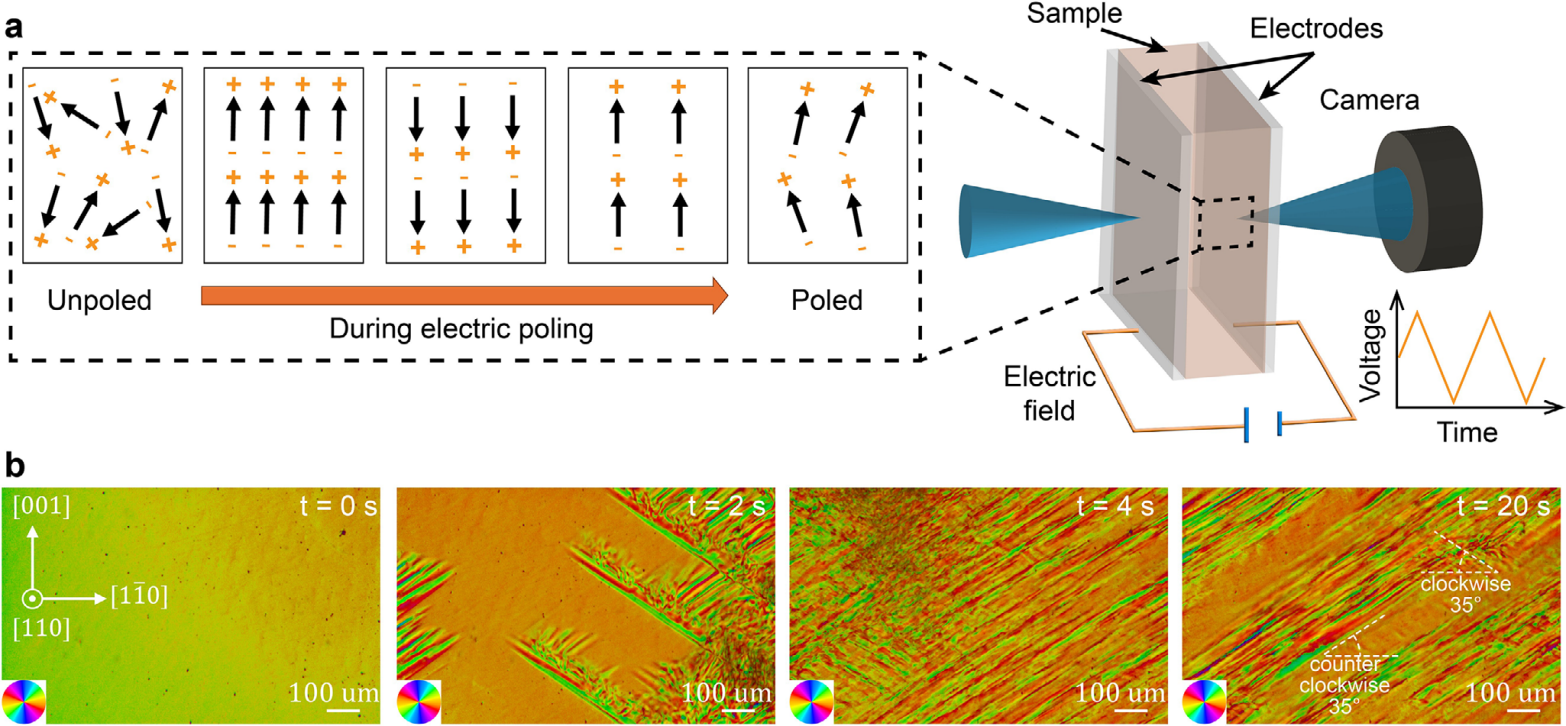
Real‐time visualization of domain wall dynamics during electrical poling. (a) Schematic of the in‐situ imaging and electrical poling synchronization scheme that enables time‐resolved imaging of ferroelectric domain‐walls during electrical poling in a PIN–PMN–PT single crystal with transparent ITO electrodes. IPOLπ images are recorded synchronously with the applied electric field, allowing direct correlation between domain‐wall evolution and instantaneous electric‐field states. (b) In situ IPOLπ images captured during AC poling on a [110]‐oriented PIN–PMN–PT single crystal, showing the time evolution of micrometer‐scale domain‐wall traces with characteristic orientations of ∼35° relative to $\left[1\bar{1}0\right]$ direction.

To visualize the real‐time evolution of ferroelectric domain structures under an applied electric field, we employed an in‐situ imaging platform based on the IPOLπ method [52, 54]. Figure 1a schematically illustrates the in‐situ imaging and electrical poling configuration, which enables continuous optical observation during the ACP of a PIN–PMN–PT single crystal coated with transparent indium tin oxide (ITO) electrodes. The AC electric field was applied through the ITO layers, while a colour camera recorded time‐resolved spectral signals arising from field‐induced changes in optical birefringence via the IPOLπ polarization‐analysis optics (details in Supplementary Note 1). Because the birefringence contrast is intrinsically linked to the local ferroelectric domain configuration, this approach provides an optical probe of birefringence changes associated with domain‐wall motion and reconfiguration inside the crystal and allows uninterrupted visualization of domain dynamics throughout the entire ACP process. The applied ACP voltage follows a triangular waveform in time, driving the ferroelectric system from an initially unpoled state toward a poled configuration through simultaneous polarization rotation and domain redistribution. A detailed description of the IPOLπ optical setup is provided in Figure S1 and in our previous publication [54].

Figure 1b shows a representative in‐situ image acquired during ACP on a [110]‐oriented PIN–PMN–PT single crystal (12 mm × 12 mm × 0.5 mm, with the [110] direction along the 0.5 mm thickness direction) coated with ∼200 nm‐thick ITO electrodes on both surfaces. The ACP was performed for 20 s at a frequency of 1 Hz, corresponding to 20 full cycles, with a peak‐to‐peak electric field amplitude of 10 kV/cm. The poling parameters are summarized in Table S1, and the detailed procedures are described in Note S1. The camera acquisition rate (100 frames per second) is sufficient to resolve the temporal evolution of domain‐wall motion within each AC cycle, while the spatial resolution (∼1 micron, 112 pixels per 100 µm) allows clear identification of individual micrometer‐scale domain walls. Multiple domain walls are observed across the field of view, oriented at approximately ±35° relative to the $\left[1\bar{1}0\right]$ crystallographic direction. This orientation is consistent with previously reported domain‐wall configurations in [110]‐orientated PIN–PMN–PT crystals orientation [31] and is independently confirmed by polarized light microscopy (PLM; Eclipse 50 iPOL, Nikon) performed on the same sample [54]. To further validate the electrical performance of the in‐house IPOLπ poling fixture, we benchmarked the poled sample properties against those obtained using the commercial poling system (aixACCT), as summarized in Table S2. The comparable electromechanical properties obtained from both methods confirm the reliability of the IPOLπ fixture and setup for in situ ACP studies.

Direct and apparent inspection of the time‐resolved image sequence reveals two main features of ferroelectric domain dynamics during the ACP process (see Video S1 for the complete evolution). First, upon application of the AC electric field, domain walls with orientations of ±35° nucleate and propagate across the imaged area, indicating a field‐driven formation and expansion of an interconnected domain‐wall network. Following an initial transient nucleation stage, the overall domain pattern becomes cycle‐stabilized, yet remains highly dynamic, with domain walls continuously responding to the oscillating external field. Second, pronounced domain‐size redistribution occurs during poling: some domains grow substantially, reaching characteristic length scales of tens to hundreds of micrometers, whereas others remain nearly unchanged or shrink. These observations indicate that IPOLπ enables real‐time visualization of optical birefringence responses associated with domain‐wall nucleation, propagation, and competitive growth processes throughout the electrical poling process, dynamical phenomena that are largely inaccessible using conventional ex situ characterization techniques.

### 2.2 Time‐Resolved Optical Orientation and Domain Dynamics During ACP

In the IPOLπ measurement, the polarization state of light transmitted through the sample is analyzed after it passes through the complete optical train. Any microstructural features that locally modify the polarization state, most notably ferroelectric domains and domain walls, produce spatial variations in optical birefringence and polarization orientation that are captured in the recorded images. To quantitatively relate these optical signals to domain evolution during AC poling, we start by analyzing the first set of key parameters extracted from the IPOLπ data: the optical orientation angle and its statistical distribution.

**FIGURE 2:**
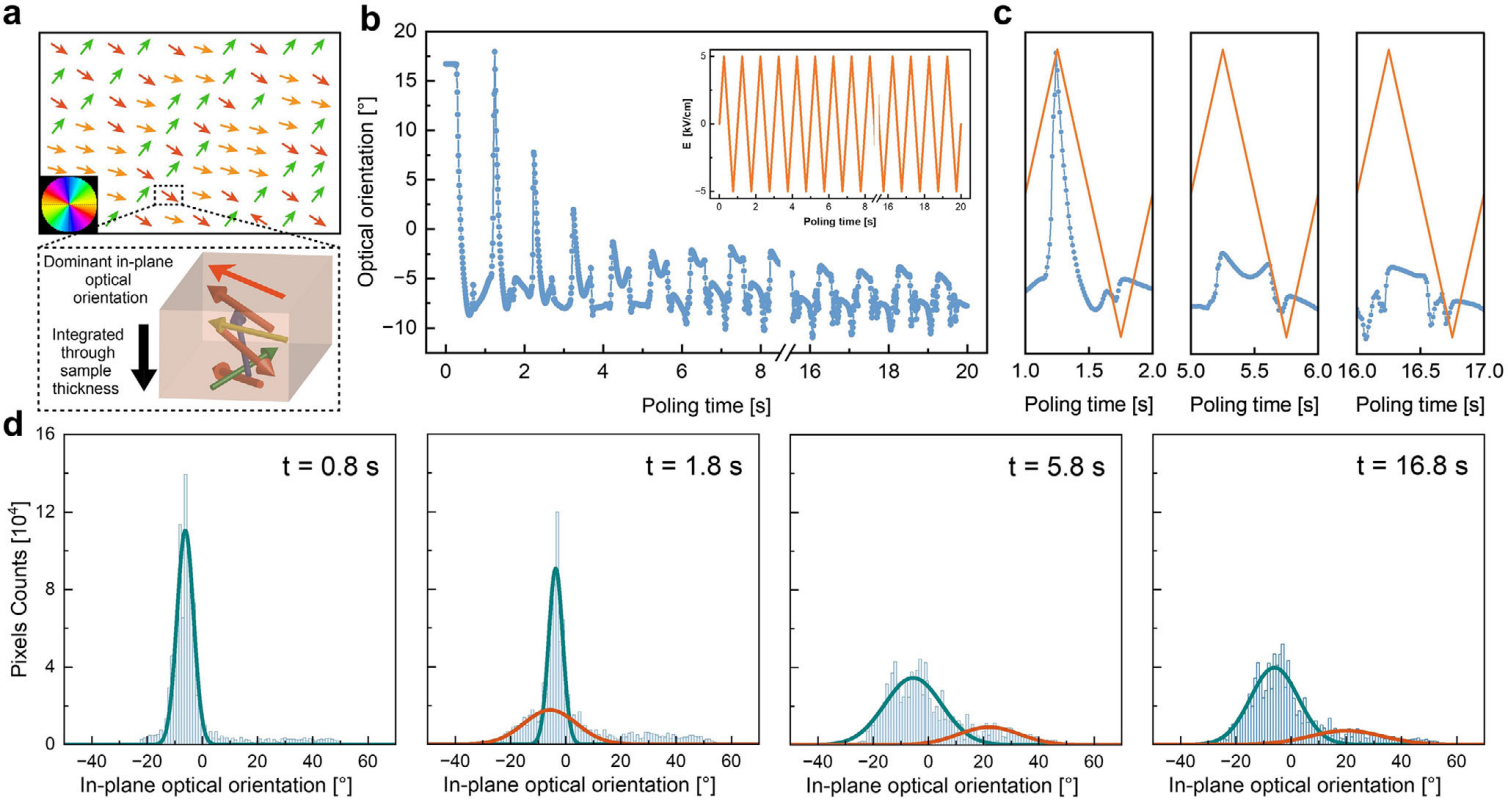
Time‐resolved optical orientation during AC poling. (a) Schematic illustration of the IPOLπ orientation‐extraction and interpretation scheme. Each pixel in the captured image is assigned an optical orientation based on its colour using a calibrated angle–colour relationship. The extracted optical orientation represents the birefringent response integrated through the sample thickness along the observation direction and reflects the dominant in‐plane optical orientation associated with the underlying domain wall configuration. (b) Time‐resolved evolution of the dominant optical orientation during the AC poling, extracted from Gaussian fitting to the orientation histograms. The inset shows the corresponding AC poling electric field applied to the sample. (c) Enlarged views of the optical orientation evolution under different AC poling cycles, showing the gradual change in optical orientation with increasing AC poling cycles. (d) Statistical distributions of the optical orientation extracted at the end of selected ACP cycles (t = 0.8 s, 1.8 s, 5.8 s, 16.8 s), fitted with Gaussian profiles (red and orange curves). The gradual emergence and stabilization of the secondary orientation peak indicate the formation of two domain wall groups at clockwise 35° and counterclockwise 35° (Figure 1b) under the applied multi‐cycle AC electric field.

As shown in Figure 2a, the optical orientation angle represents the dominant in‐plane birefringent response integrated along the optical path ([110] direction in this study) at each image pixel. The RGB (i.e., red, green, and blue) response at each pixel was calibrated using a polycarbonate reference sample rotated through known in‐plane orientations to construct a color–orientation mapping [54]. In this convention, the horizontal in‐plane direction was defined as 0°, and the optical orientation angle increases counterclockwise with a periodicity of 180°. Because the IPOLπ signal is accumulated through the full sample thickness, this optical orientation does not directly correspond to the 3D geometric orientation of individual domains or domain walls. Instead, variations in the optical orientation reflect collective changes in domain‐wall configurations and their relative population within the probed volume. Accordingly, the present IPOLπ measurements primarily resolve micrometer‐scale domain‐wall traces and mesoscale domain‐wall textures. Sub‐resolution features, including nanoscale domain‐wall internal structures, local defects, and very fine domain variants, are not individually resolved but may still influence domain‐wall mobility and switching kinetics and therefore contribute indirectly to the measured thickness‐integrated optical response. By computing the optical orientation angle for every pixel in each time‐resolved frame, we obtain a spatially resolved map of the evolving domain‐wall orientations throughout the ACP process. Furthermore, as IPOLπ detects the polarization state of transmitted light, possible Pockels‐ and Kerr‐type electro‐optic effects under applied fields may contribute a field‐synchronized background [31, 55].

To track the global domain response, we construct optical orientation histograms from all pixels within the field of view for each time frame. Each histogram is fitted using Gaussian functions to extract the dominant orientation features, following standard directional statistics approaches for axial (180°‐periodic) angular data [56]. The peak position of the Gaussian fit serves as a quantitative descriptor of the prevailing domain‐wall orientation, while the distribution width reflects the degree of orientational dispersion and alignment uniformity. Tracking the evolution of these fitted parameters enables a time‐resolved, statistical description of domain dynamics under the applied AC electric field.

We note here that the IPOLπ system has been calibrated to isolate the optical response originating solely from the ferroelectric crystal. In particular, we find that the presence of ITO electrodes introduces wavelength‐dependent transmission variations that influence the spectral encoding of optical polarization information. This calibration ensures that the reported orientation values faithfully reflect domain‐induced birefringence rather than artifacts from the electrode layers(see Figure S3).

Figure 2b summarizes the time evolution of the dominant optical orientation extracted from Gaussian fits, together with the applied AC electric field waveform (inset). During the first four AC cycles, the optical orientation exhibits pronounced fluctuations, indicative of an actively evolving domain‐wall configuration that strongly responds to the alternating electric field. With continued cycling, the response progressively stabilizes: beyond approximately four cycles, the dominant optical orientation remains confined within a narrow angular range of ∼10°, while still exhibiting a periodic modulation synchronized with the applied field (Figure 2c). This behavior demonstrates that ACP does not simply drive the system into a static, field‐independent domain state; instead, the domain configuration becomes cycle‐stabilized yet remains dynamically responsive to the electric field. This cycle‐dependent evolution is consistent with prior reports on ACP‐induced modifications of domain structures and electromechanical properties [12, 14, 46, 57].

Further insight is obtained from statistical analysis of the optical orientation distributions at selected times during ACP (Figure 2d). The histograms initially exhibit a single dominant peak, which evolves into a clear double‐peak structure during the first four AC cycles. The secondary peak gradually emerges and shifts before both peaks stabilize at approximately −7° (dominant peak) and 23° (secondary peak), corresponding to the final domain configuration after ACP. This evolution mirrors the in‐plane domain‐wall patterns observed in the time‐resolved IPOLπ images (Figure S3), where distinct domain‐wall traces progressively develop and subsequently display a repeatable, field‐synchronized response. The higher‐intensity peak corresponds to the dominant set of clockwise ∼35° domain‐wall traces visible in the images (Figure 1b), whereas the lower‐intensity peak appears only when a smaller population of counterclockwise ∼35° traces are present within the field of view. Together, these results reveal that the ferroelectric domain response under ACP is governed by a periodic, field‐driven reconfiguration established after several cycles, rather than by a single irreversible transition into a static domain arrangement.

### 2.3 Reversible, Dynamic, and Selective Domain‐Wall Response Within a Representative AC Cycle

We now focus on the dynamically steady stage of AC poling, where the ferroelectric domain configuration becomes cycle‐stabilized while remaining responsive to the applied electric field. During the later stages of ACP (for example, 16–17 s in Figure 2c), the dominant optical orientation exhibits a periodic modulation that closely follows the AC electric field, yet remains confined within a narrow angular window. This behavior indicates that the system has reached a repeatable in‐plane orientation state that is dynamically maintained rather than frozen. To further interrogate the microscopic origin of this dynamic response, we examine additional optical observables extracted from the in‐situ IPOLπ measurements.

**FIGURE 3:**
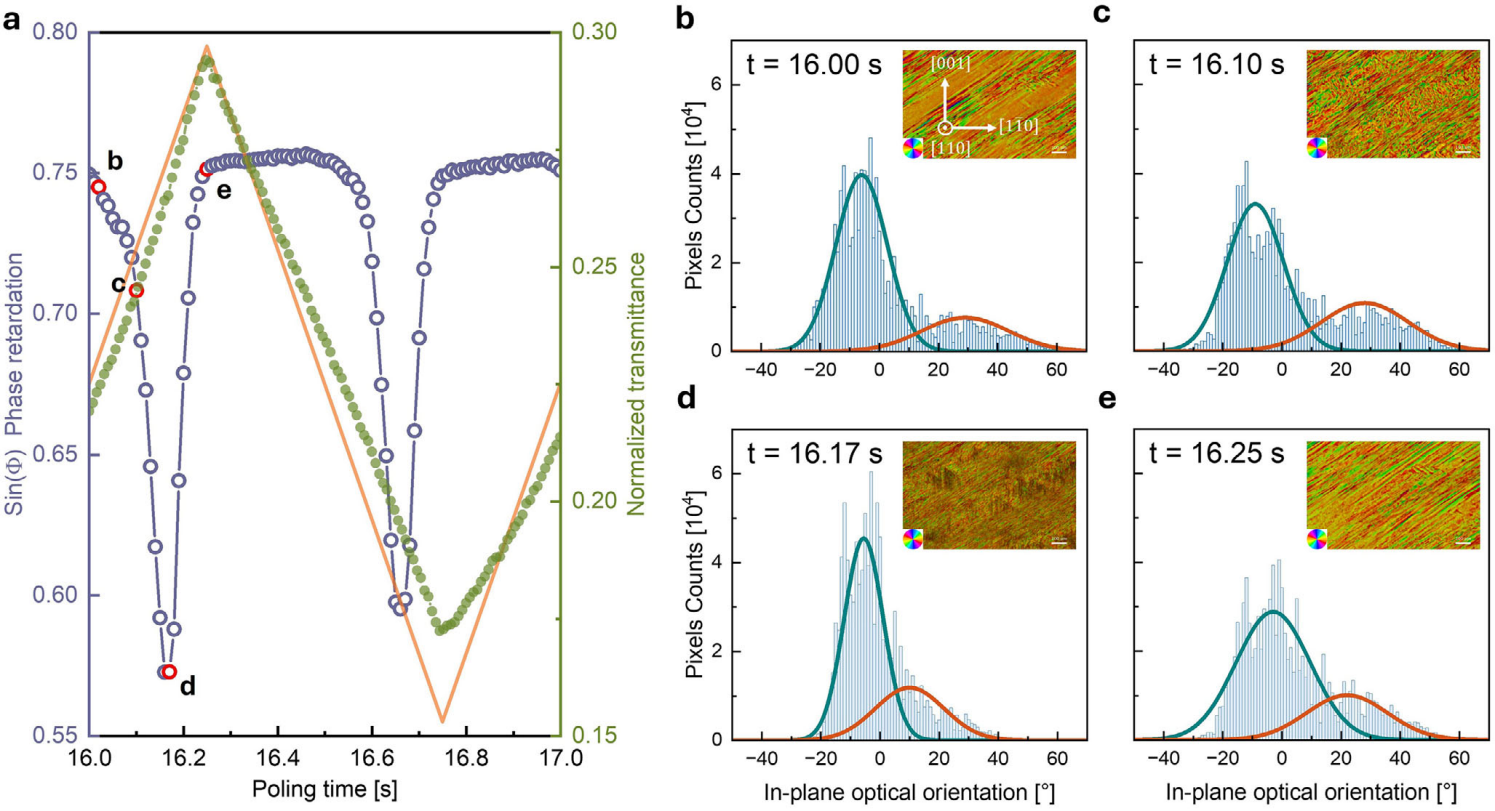
Dynamic optical response during the representative AC poling cycle. (a) Time‐resolved response of the phase retardation signal ($\sin(\phi_{S})$) and normalized transparency during a representative AC poling period (16–17 s), together with the applied AC electric field (orange line). (b–e) Optical orientation distribution histograms at selected time points within the same AC cycle (t = 16.00 s, 16.10 s, 16.17 s, and 16.25 s). Insets show the corresponding camera images. The sharp change in $\sin(\phi_{S})$) coincides with the shift of the secondary orientation peak in the histograms and a redistribution of the counterclockwise ∼35° domain‐wall traces, while the dominant clockwise ∼35° domain‐wall population remains unchanged.

We first analyze the phase‐retardation‐related signal, $\sin(\phi_{S})$, which is extracted from the IPOLπ image brightness and reflects the phase retardation induced by sample birefringence, where $\phi_{S}$ denotes the physical retardation accumulated through the crystal thickness. Although IPOLπ does not provide the absolute value of $\phi_{S}$, relative changes in $\sin(\phi_{S})$ directly capture variations in birefringence associated with domain reconfiguration. Within a single AC cycle, the phase‐retardation signal exhibits two pronounced and sharp transitions, occurring once in each half‐cycle after each zero crossing as the field magnitude increases from zero to the peak value (Figure 3a). Given that the dominant in‐plane optical orientation remains confined within a narrow angular window, these abrupt retardation changes are attributed primarily to the field‐driven reconfiguration of the thickness‐integrated birefringent response associated with domain‐wall motion and rearrangement along the electric‐field direction. Importantly, this characteristic response is highly reproducible across successive cycles once the dynamically steady state is established (Figure S4), confirming that the observed birefringence changes are intrinsic to the stabilized ACP regime rather than transient artifacts.

As a complementary probe, we next examine the through‐thickness optical transmission of the crystal, which serves as a third metric of domain dynamics. To isolate this effect, all polarization‐selective optical components were removed from the IPOLπ setup, and the transmitted light intensity was recorded in situ using a monochrome camera. Here, the transmitted intensity is reported in a normalized form using the values measured before poling (unpoled state) and after poling (final poled state) as reference points. The normalized transmission exhibits clear periodic modulation within a single AC cycle, with a frequency identical to that of the applied electric field (Figure 3a). The reversible increases and decreases in transmission also indicate cyclic rearrangements of the domain‐wall structure along the thickness direction, consistent with a field‐driven modulation of internal scattering and birefringence during each AC cycle.

To determine whether this dynamic response involves all domain walls or only specific subsets, we perform a detailed analysis within a single representative cycle (16–16.25 s), as shown in Figure 3b–e. Over this interval, the dominant optical orientation, corresponding to the main peak in the orientation histograms, exhibits only minor variation, remaining within a narrow angular range from approximately −4° to −12° (Figures 2c and 3b–e). In contrast, the secondary orientation peak shows pronounced and reversible shifts, moving from ∼30° to ∼10° before returning to its original position. The relative population associated with the secondary peak changes, indicating a reversible redistribution rather than a uniform shift of the entire domain ensemble. This behavior is corroborated by the time‐resolved IPOLπ images, which reveal a reversible redistribution of the counterclockwise ∼35° domain‐wall traces, while the dominant clockwise ∼35° domain‐wall network remains largely unchanged. These observations link the sharp drop and recovery in $\sin(\phi_{S})$ to a selective, field‐synchronized reconfiguration of the counterclockwise 35° domain‐wall group, while the dominant clockwise 35° group remains largely stationary. Upon completion of one full AC cycle, the domain configuration nearly returns to its initial state at 16 s, directly demonstrating a reversible, field‐synchronized domain‐wall motion.

Taken together, the direct imaging observation, optical orientation histogram, phase‐retardation, and transmission results establish that ACP induces a reversible and selective domain‐wall response within each electric‐field cycle. In this dynamically steady regime, the dominant clockwise ∼35° domain‐wall population forms a stable backbone of the domain structure, whereas the secondary counterclockwise ∼35° domain walls undergo reversible reconfiguration synchronized with the applied AC field. This selective dynamic behavior highlights the nonequilibrium yet highly ordered nature of the ACP‐stabilized domain state.

### 2.4 Optical Response and Domain Dynamics During DCP

**FIGURE 4:**
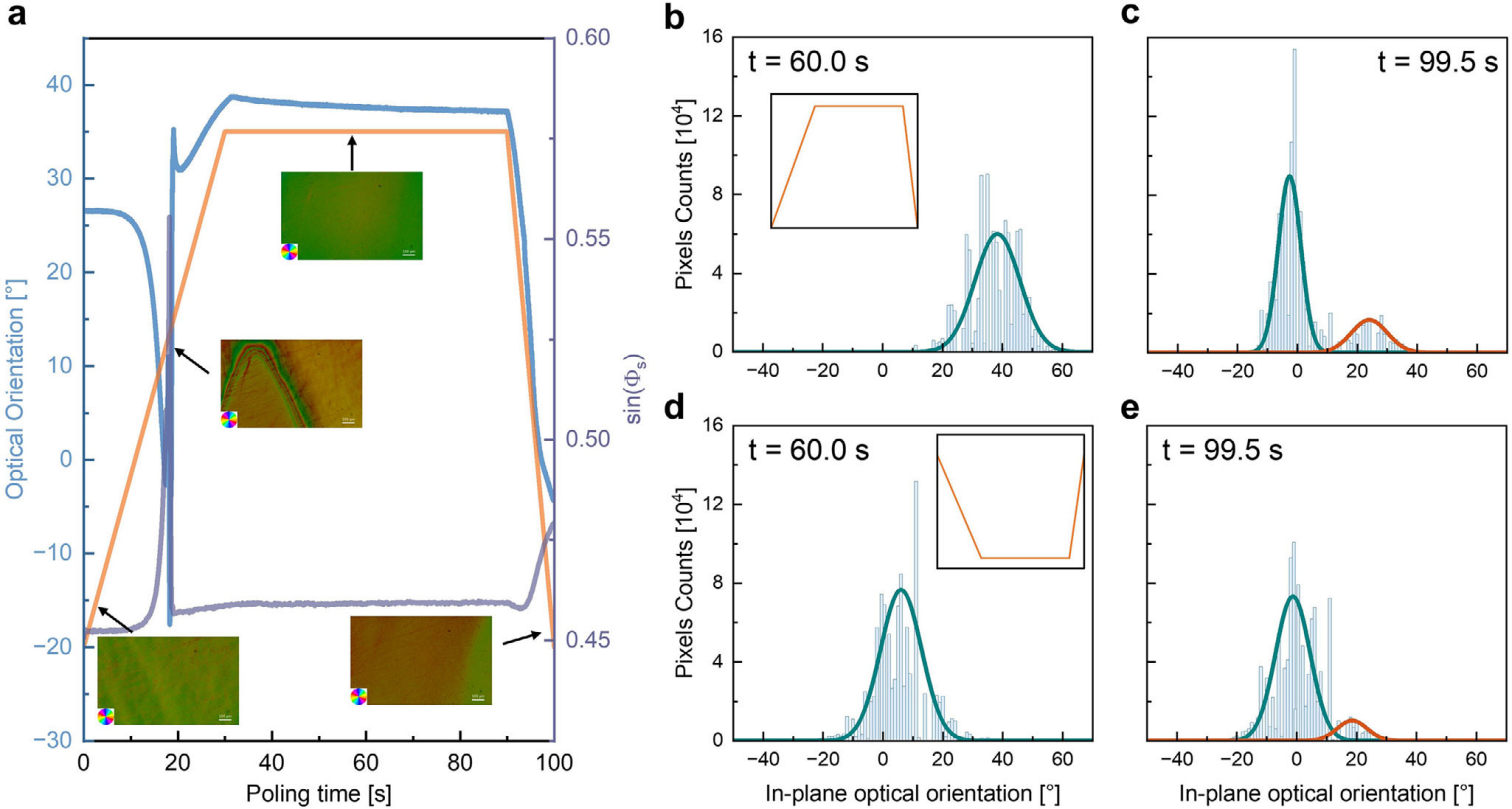
Time‐resolved optical response during DC poling. (a) Time‐resolved response of the optical orientation (blue), phase retardation signal ($\sin(\phi_{S})$) (purple) and applied electric field (yellow) during the DC poling process. Insets show representative captured images at selected time points during DCP, including the initial state, field‐increasing stage, steady DC‐field holding state, and final state. Sharp changes in both optical signals occur during the initial increase and final decrease of the electric field, whereas both signals remain nearly constant under the steady DC field. (b–e) Statistical distributions of cross‐plane optical orientation at representative moments (t = 60.0 s and 99.5 s) under opposite DC field directions. Insets show the corresponding DC electric field. The reversal of the DC field direction leads to an inversion of the orientation distribution during the poling process. Both cases exhibit similar main peak positions and a small secondary peak, showing comparable final poling states despite the opposite field directions.

To contrast the domain evolution induced by AC poling, we performed in‐situ DC poling measurements on the same [110]‐oriented PIN–PMN–PT single crystal. A unidirectional DC electric field with a peak magnitude of 5 kV/cm was applied for a total duration of 100 s, as shown in Figure 4a. Upon the initial rise of the DC field, both the optical orientation and the phase retardation signals exhibit sharp and abrupt changes. This response reflects rapid domain reorientation driven by the unidirectional electric field and is consistent with established DC‐field‐induced switching behavior in relaxor ferroelectrics [40]. The observed response also mirrors the field‐driven transitions captured during the rising segments of the ACP cycles, confirming the consistency between DCP and ACP measurements.

Once the DC field reaches its peak value and is held constant, both optical signals remain essentially unchanged, indicating that the domain configuration stabilizes under the static electric field. In contrast to ACP, no periodic modulation or dynamic response is observed during this holding stage, demonstrating that the domain structure under DCP rapidly evolves into a static configuration. When the DC field is reduced and eventually removed, the optical signals exhibit only a slight relaxation and do not return to their initial pre‐poling values. This residual offset is probably attributed to incomplete back‐switching or back‐tilting of domains after the removal of the external electric field, reflecting the partially irreversible nature of DC‐induced domain reorientation.

To further validate the robustness of these observations, we repeated the DCP measurements with the DC electric field applied in the opposite direction, as shown in Figure 4b–e. During the holding stage of the DC field (approximately 60 s into the poling process), the optical orientation histograms exhibit markedly different peak positions for opposite field polarities: a dominant peak near ∼37° under the positive field and near ∼5° under the reversed field (Figure 4b,d). These polarity‐dependent orientation states confirm that the instantaneous domain configuration during DCP is strongly dictated by the direction of the applied DC electric field. In contrast, after the DC field is fully removed, the final optical orientation distributions converge to similar profiles for both field directions, characterized by a main peak near −10° and a much weaker secondary peak around ∼20° (Figure 4c,e). This final distribution closely resembles that obtained after ACP, albeit with a significantly reduced secondary peak intensity in the DCP case. The convergence of the final domain states indicates that, despite polarity‐dependent transient configurations during DCP, the remanent domain orientation after field removal is largely insensitive to the electric field direction. Compared to ACP, DCP therefore yields a more static and less heterogeneous domain configuration, lacking the pronounced reversible secondary domain population stabilized under cyclic electric fields.

### 2.5 Optical Response and Domain Dynamics During EDP

**FIGURE 5:**
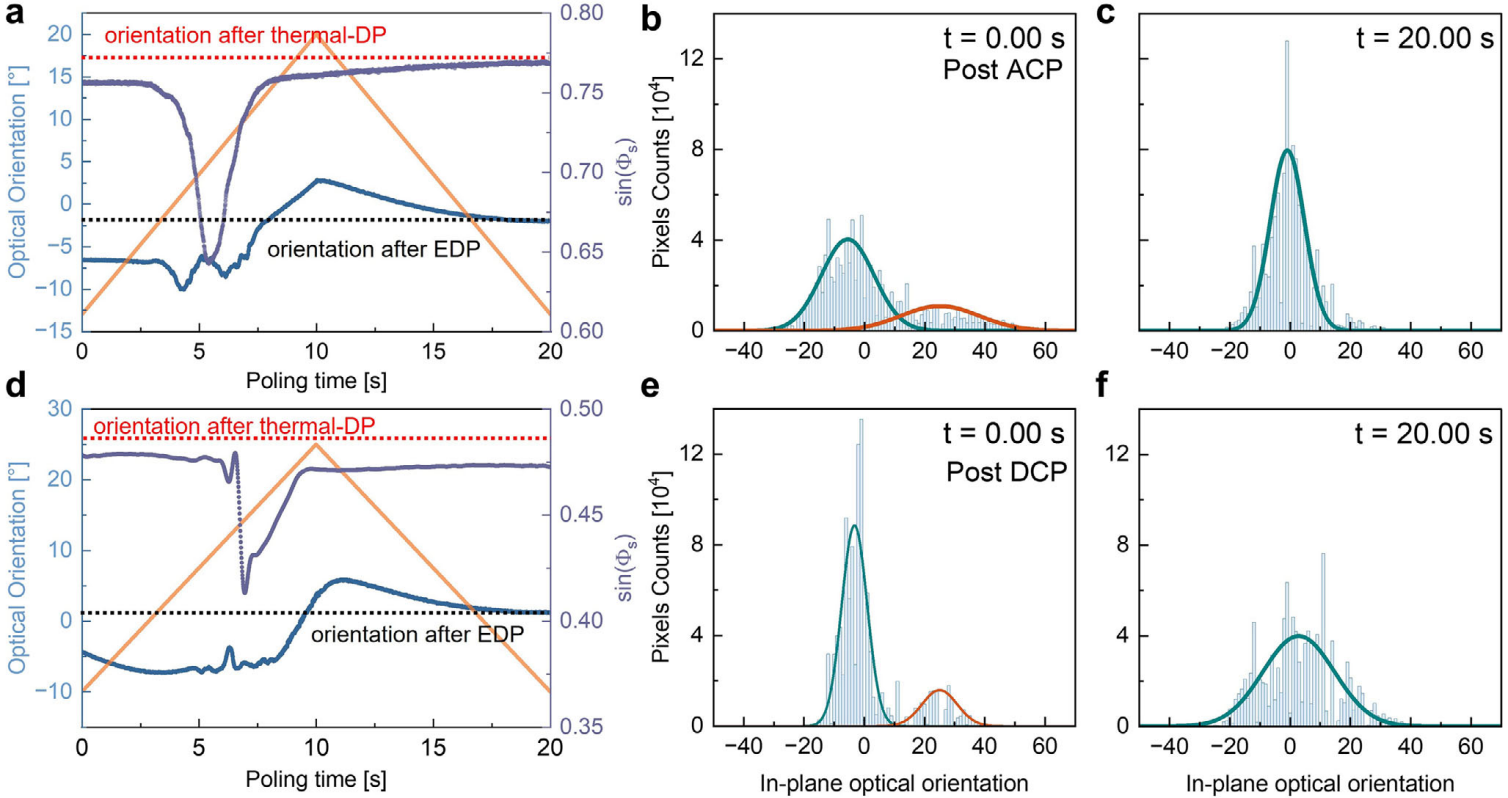
Time‐resolved optical response during electrical depoling. (a, d) Time‐resolved response of the optical orientation (blue), phase retardation signal ($\sin(\phi_{S})$) (purple), and applied electric field (yellow) during the electrical depoling (EDP) process for AC poled (a) and DC poled (d) samples. Dashed lines mark the final orientation states after EDP and after thermal depoling (thermal‐DP). (b, c, e, f) Statistical distributions of cross‐plane optical orientation at representative moments (t = 0.0 s and 20.0 s), fitted with Gaussian profiles. The disappearance of the secondary peak and the convergence of the main peak close to zero degrees indicate a relaxation of the domain alignment during electrical depoling.

Electrical depoling experiments were performed on both [110]‐oriented AC‐poled and DC‐poled PIN–PMN–PT samples, using a low‐frequency AC electric field with a peak amplitude of 5 kV/cm, following protocols reported previously [22]. The EDP parameters were selected and confirmed by measurements of the samples’ dielectric and piezoelectric properties (Table S2). In this process, depolarization is driven by a controlled electric‐field excursion at room temperature, allowing relaxation of the existing domain configuration without thermal treatment. Figure 5a–c summarizes the EDP optical responses for the AC‐poled sample, while Figure 5d–f presents the corresponding results for the DC‐poled sample.

In both cases, the time‐resolved phase‐retardation signal exhibits a sharp change during the rising stage of the depoling field, followed by a return toward a value close to the initial level once the field is reduced. This behaviour mirrors that observed during ACP and DCP, indicating that domain reconfiguration during EDP is likewise primarily activated during the increasing‐field segment. Despite the different initial poling histories, the optical orientation histograms for both samples evolve in a similar manner during EDP: the secondary orientation peak progressively disappears, and the distributions converge toward a single dominant peak (Figure 5b,c,e,f). After completion of the EDP process, the final optical orientation stabilizes near ∼0° for both AC‐poled and DC‐poled samples. Repeated measurements across multiple specimens confirm the reproducibility of this convergence. The emergence of a single‐peak orientation distribution and the convergence to a common final orientation indicate a substantial reduction of internal strain and a relaxation of domain alignment during electrical depoling. Notably, the post‐EDP optical orientation and phase‐retardation signals differ from those obtained after thermal depoling, demonstrating that EDP and thermal depoling access distinct relaxed domain states.

While the statistical optical metrics converge after EDP, direct IPOLπ imaging reveals clear differences in the mesoscale domain‐wall patterns that depend on the prior poling history. For the AC‐poled sample, the prominent domain‐wall traces formed during ACP are largely suppressed following EDP, although a small fraction of residual domain‐wall features remains visible. In contrast, the DC‐poled sample exhibits the emergence of additional ∼35° domain‐wall patterns after EDP, with characteristic length scales comparable to those observed during ACP (Figure S10). These image‐level direct observations indicate that EDP relaxes the domain structure but does not erase all poling‐induced domain‐wall features in the same manner as thermal depoling.

The contrasting responses can be understood by considering the combined DCP–EDP sequence as an effective AC‐like field cycle occurring over a longer time scale, which promotes the formation of larger‐scale, multidirectional domain‐wall networks in the DC‐poled sample. To further validate the relaxed nature of the post‐EDP state, we applied a subsequent DCP using the same field direction as that of the EDP cycle. In contrast to the pronounced reconfiguration observed when the post‐EDP DCP is applied with reversed field direction, the same‐polarity DCP induces only a delayed and gradual evolution of the optical orientation without the abrupt transition, and the phase retardation signal ($\sin(\phi_{S})$) remains stable during the whole process (Figure S11). These results show that the EDP process removes most of the nonequilibrium driving force, leaving the system in a near‐relaxed state with slow residual dynamics. Moreover, repeated multi‐cycle EDP–DCP–EDP–DCP tests reveal that the domain evolution induced by DCP progressively resembles ACP‐like behaviour (Figure S12), suggesting a gradual transition from single‐direction domain alignment toward multidirectional, dynamically reconfigurable domain states under alternating electrical histories.

Collectively, these results demonstrate that electrical depoling drives distinct initially poled states toward a common, relaxed orientation distribution, while preserving pronounced differences in mesoscale domain‐wall organization that encode the prior poling history. This decoupling between global orientation relaxation and local domain‐wall topology highlights the unique role of electrical depoling in tuning nonequilibrium ferroelectric domain states.

### 2.6 Variation of Domain Size and Homogeneity under Different Poling Conditions

The ∼1 µm spatial resolution of the IPOLπ technique enables direct interrogation of domain size and mesoscale homogeneity under different electrical poling conditions. This capability is particularly relevant given the long‐standing debate regarding the microstructural origin of ACP‐enhanced electromechanical performance: some studies attribute the enhancement to domain refinement and increased domain‐wall density [6, 7, 29], whereas others associate it with domain coarsening [30, 31, 32, 33] and the formation of larger‐scale domain structures. Our in‐situ observations provide a unified framework to reconcile these seemingly contradictory interpretations.

Under ACP, time‐resolved imaging reveals pronounced domain‐size redistribution rather than a monotonic refinement process. As shown in Figure 1b, certain domains grow substantially during cycling, reaching characteristic in‐plane length scales of tens to hundreds of micrometers, while others shrink or remain relatively unchanged. This heterogeneous evolution is accompanied by a clear broadening of the optical orientation distributions: the histograms evolve from an initially narrow single‐peak feature to a stabilized double‐peak structure (Figure 2d). The emergence of a secondary orientation population and the increase in histogram width (e.g., full width at half maximum) indicate enhanced orientational dispersion and reduced alignment uniformity under ACP. Consistent with this picture, the normalized optical transmission decreases gradually over the 20 ACP cycles, from approximately 70% to 25% (Figure S5). Because this transmission integrates optical scattering and birefringence from all domains through the full sample thickness, the monotonic decrease reflects a progressive increase in through‐thickness domain‐wall complexity and mesoscale inhomogeneity.

In contrast, DCP is characterized by a single, field‐driven reconfiguration event. Both optical orientation and phase‐retardation signals exhibit sharp changes only during the initial rise of the DC field, followed by a stable response once the field reaches its peak (Figure 4a). This behavior indicates that ACP and DCP are governed by the same underlying field‐activated mechanism, with the key distinction being the presence or absence of repeated bipolar cycling. In this sense, DCP can be viewed as analogous to a single ACP ramp segment (from zero to peak field) without field reversal or repeated activation. Correspondingly, the optical orientation histograms in DCP remain relatively narrow and evolve toward a dominant single peak, with only a weak secondary population at the end of poling (Figure 4c,e). Time‐resolved images show no persistent large‐scale (≫1 µm) domain‐wall traces beyond the brief transition period; instead, the images exhibit smooth spatial color gradients with minimal sharp contrast (Figure S8). This behavior is consistent with a more homogeneous domain configuration under unidirectional DC fields, with reduced visibility of mesoscale domain‐wall textures.

Electrical depoling further highlights the strong dependence of mesoscale domain structure on poling history. Although the optical orientation distributions of both AC‐ and DC‐poled samples converge toward a single dominant peak after EDP, direct imaging reveals markedly different post‐EDP domain patterns. In AC‐poled samples, the large‐scale domain‐wall traces formed during ACP are largely suppressed following EDP, whereas DC‐poled samples develop additional ∼35° domain‐wall patterns with length scales comparable to those observed during ACP (Figure S10). Moreover, when DCP is applied after EDP, ACP‐like large‐scale domain‐wall patterns re‐emerge during the field‐increasing stage (around ∼20 s; Figure S12). This behavior supports the interpretation that the combined EDP–DCP sequence effectively introduces a bipolar field excursion, rendering it mechanistically closer to an ACP cycle than to a purely unidirectional DCP process.

**FIGURE 6:**
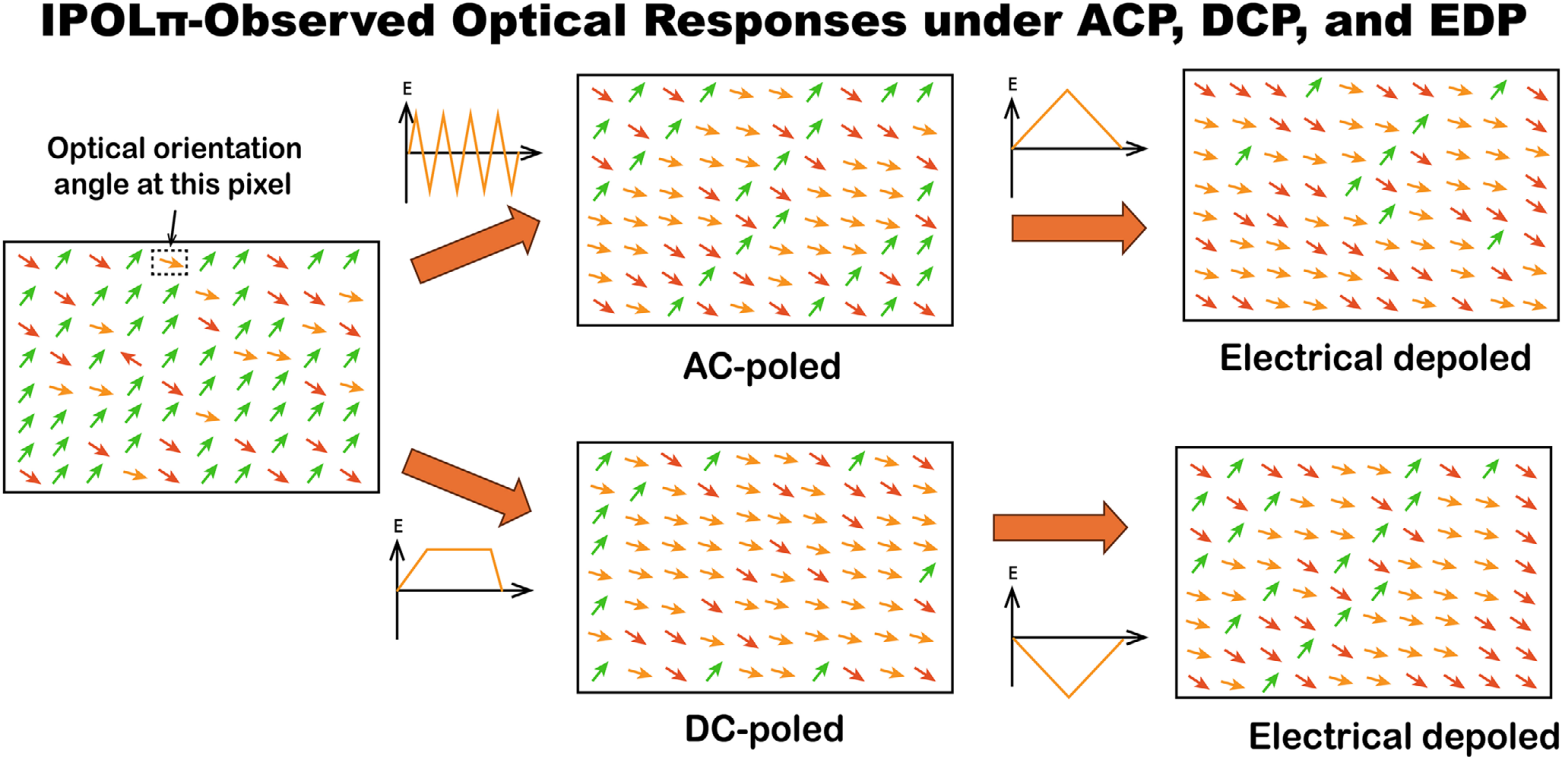
Schematic summary of IPOLπ‐observed optical‐orientation evolution during ACP, DCP, and EDP. The colored arrows represent local optical orientations extracted from the thickness‐integrated birefringence response rather than true 3D ferroelectric polarization vectors or fully reconstructed domain variants. ACP produces a heterogeneous and dynamically responsive optical‐orientation distribution, whereas DCP results in a more homogeneous orientation state with reduced mesoscale texture. During EDP, both AC‐poled and DC‐poled samples relax toward a more converged global optical‐orientation distribution, but their mesoscale optical textures remain history‐dependent: ACP‐induced optical traces are largely suppressed after EDP, whereas DCP‐poled samples can develop additional domain‐wall‐related optical patterns. The schematic summarizes the qualitative optical responses inferred from the IPOLπ measurements.

To provide an integrated visual summary of these protocol‐dependent optical responses, Figure 6 schematically shows the IPOLπ‐derived picture developed in this section. Taken together, these results demonstrate that ACP‐induced performance enhancement is accompanied by the stabilization of larger‐scale, spatially heterogeneous domain‐wall textures, rather than by a simple refinement into finer, static domains. Repeated bipolar field cycling promotes domain redistribution and orientational dispersion across multiple length scales, whereas unidirectional DC poling favors a more homogeneous and more weakly textured domain configuration. EDP further introduces a history‐dependent relaxation pathway: although the global optical‐orientation distributions converge after EDP, AC‐poled samples mainly show suppression of ACP‐induced optical traces, and DC‐poled samples can develop additional domain‐wall‐related optical patterns. This distinction provides a coherent explanation for the divergent interpretations in the literature and underscores the importance of dynamic cycling history in shaping ferroelectric domain architecture.

## 3 Discussion

In this work, we establish in‐situ IPOLπ imaging as a direct and quantitative approach to visualize ferroelectric domain‐wall dynamics related to birefringence responses under ACP, DCP, and EDP. By tracking optical orientation and phase‐retardation responses in real time, we access domain wall nucleation, motion, and reconfiguration during active electrical driving, processes that have largely remained inferred from pre‐/post‐poling measurements or bulk‐averaged properties in prior studies [14, 58]. This capability resolves a central limitation in the ACP/DCP literature, where competing interpretations such as domain refinement versus domain coarsening have coexisted without direct evidence of the underlying dynamics.

Our in‐situ results reveal that ACP drives a two‐stage domain evolution: an early transient regime marked by large fluctuations and domain wall nucleation, followed by a dynamically steady regime in which a cycle‐stabilized domain wall network remains reversibly responsive to the applied field. The emergence and stabilization of two domain wall populations, together with periodic retardation jumps synchronized with each AC half‐cycle, demonstrate that ACP does not produce a single static “optimized” domain state. Instead, it establishes a field‐synchronous, low‐strain domain configuration that remains dynamically active beyond initial poling [12, 14, 39, 58]. This directly supports domain‐wall‐mediated reversibility as the defining kinetic feature of ACP and explains the strong dependence of ACP outcomes on cycle number, waveform, and frequency reported across relaxor‐PT systems [26, 40, 46].

In contrast, DCP produces a fundamentally different kinetic pathway. Domain reconfiguration occurs predominantly during the field ramp, after which both optical orientation and phase retardation stabilize, indicating completion of unidirectional alignment. Although reversing the DC field changes the transient orientation state, the final remanent configuration after field removal is similar, highlighting that DCP primarily suppresses domain wall mobility once alignment is achieved. Compared to ACP, DCP yields fewer visible mesoscale domain wall features, indicating a more homogeneous but less dynamically adaptable domain structure. The key distinction between ACP and DCP, therefore, lies not in the final average orientation alone, but in the degree of sustained domain wall mobility enabled by the driving waveform.

Electrical depoling further clarifies the relationship between reversibility, strain relaxation, and domain history. EDP reproducibly drives both AC‐ and DC‐poled samples toward a common relaxed orientation distribution, confirming its effectiveness as a non‐thermal depolarization route. However, direct imaging reveals that mesoscale domain textures remain strongly dependent on the initial poling history, in contrast to thermal depoling. This demonstrates that EDP relaxes polarization and internal strain through field‐driven domain wall rearrangement rather than through thermodynamic reinitialization. Consequently, EDP should be viewed as a distinct relaxation pathway that neutralizes macroscopic poling indicators while preserving hidden structural memory that can influence subsequent switching behavior.

Taken together, these findings provide a unified framework for understanding how different electrical poling conditions sculpt ferroelectric domain architectures across length scales. ACP promotes heterogeneous, large‐scale domain textures stabilized by cyclic driving; DCP favors rapid alignment with limited sustained domain wall activity; and EDP drives convergence of global orientation while retaining mesoscale history. This resolves long‐standing ambiguities in the interpretation of ACP‐induced performance enhancement and highlights the central role of domain wall kinetics, rather than static domain morphology, in governing functional responses.

Several limitations of the present study should be noted. IPOLπ requires sufficient optical transparency of both the crystal and electrodes, restricting its applicability to transparent ferroelectrics. The extracted optical orientation represents a birefringence‐weighted, through‐thickness average and does not uniquely resolve three‐dimensional domain variants. In addition, the present work focuses on a single crystallographic orientation and low‐frequency driving fields. Extending this approach to different cuts, higher frequencies, and varied waveforms will be necessary to generalize the identified kinetic regimes and to establish quantitative links to device operating conditions.

Despite these limitations, the ability to directly image domain‐wall‐related optical signatures in real time, which indicates the domain wall kinetics in real‐time, represents a significant advance. By systematically combining orientation‐dependent measurements across different geometries and field protocols, IPOLπ offers a pathway toward reconstructing 3D ferroelectric domain dynamics. More broadly, this work demonstrates that dynamic, history‐dependent domain‐wall behavior, rather than static domain refinement alone, provides the key microscopic basis for engineering ferroelectric functionality under complex electrical driving.

## 4 Conclusion

In summary, in situ IPOLπ imaging provides a direct and quantitative approach to visualize ferroelectric domain‐wall‐related birefringence dynamics during alternating‐current poling, direct‐current poling, and electrical depoling in PIN–PMN–PT single crystals. Real‐time tracking of optical orientation and phase‐retardation responses shows that these electrical driving protocols follow distinct kinetic pathways, rather than a common domain evolution toward different final states. Alternating‐current poling stabilizes a cycle‐responsive and spatially heterogeneous domain‐wall network, whereas direct‐current poling produces a more homogeneous domain configuration with limited sustained domain‐wall mobility after the initial field‐driven reconfiguration. Electrical depoling drives convergence of the global orientation distribution while preserving mesoscale domain textures that remain dependent on the initial poling history. Taken together, these results support the important role of domain‐wall kinetics, rather than static domain morphology alone, as the key factor governing ferroelectric response under complex electrical driving and provide a dynamic framework for rational domain‐wall engineering in ferroic materials.

## 5 Experimental Section

### 5.1 Sample Preparation

The [110]‐oriented PIN (24%)–PMN–PT single crystals were obtained from CTS Corp., IL, USA, and diced into plate‐like pieces (12 mm × 12 mm × 0.5 mm). Both top and bottom surfaces were polished along the [110] direction; the final thickness is ∼0.34 mm. Transparent indium tin oxide (ITO) electrodes with 200 nm thickness were deposited on both top and bottom surfaces (12 mm × 12 mm) with a size of 12 mm × 10 mm via sputter deposition. The ITO sputtering was performed at a chamber pressure of ∼2 mTorr and a power of 100 W (Kurt J. Lesker PRD036170), using argon and oxygen gas flows of 60 sccm and 2 sccm, respectively. The samples were mounted on a rotating holder with electrical tape, covering only the edges to minimize obstruction—no additional shadow mask was applied. After 40 minutes of sputtering, a 200 nm‐thick ITO film was obtained. The film thickness was measured using a profilometer (Bruker Dektak XT).

### 5.2 IPOLπ Setup and System Calibration

The IPOLπ system was developed based on previous studies [50, 51, 52, 53, 59, 60, 61], and the optical setup is shown in Figure S1. A broadband unpolarized white light source (LCS‐6500‐65‐22, Mightex) first passes through a vertically oriented linear polarizer (10LP‐VIS‐B, Newport) and a fixed quarter‐wave plate (10RP54‐1B, Newport). The fast and slow axes of the wave plate are aligned at 45° relative to the polarization axis, generating circularly polarized light. The circularly polarized light then transmits through the ferroelectric specimen and a stack of z‐cut quartz plates (models WQZ2525 and WQZ2501, Knight Optical, UK) with a total thickness of 6 mm (two 2.5 mm and one 1.0 mm plate). Due to the wavelength‐dependent optical rotary dispersion of quartz, different spectral components experience different rotation angles while propagating through the stack, forming a wavelength‐dependent spectral fan. This controlled dispersion encodes the polarization state into colour information across the visible spectrum, providing a continuous 180° colour variation corresponding to the optical orientation of the crystal. After transmission through the sample, the light passes through a horizontally oriented linear polarizer (10LP‐VIS‐B, Newport). The resulting transmitted light produces a colour pattern directly dependent on the local birefringence and optical orientation of the specimen. Figure S2 shows a photo of the in‐situ IPOLπ setup with the poling components. More information about the system setup is described in Notes S1 and S2.

Calibration was performed to establish quantitative mappings between the image colour and the optical properties of the specimen. A birefringent reference plate with uniform retardation was mounted on a rotation stage and rotated from 0° to 180° while recording colour images under the same optical configuration. The hue values extracted from each frame were fitted to a smooth periodic function to create a hue–orientation calibration curve, which was subsequently used to convert the colour hue of each experimental image into an optical orientation angle (θ). Retardation (Δ) calibration was performed at a fixed azimuth by varying the optical path difference using reference retardation plates and recording the corresponding intensity changes. The measured intensity–retardation relation was fitted to a monotonic function, enabling Δ extraction independent of orientation. During experiments, both θ and Δ were reconstructed from each colour frame using the two calibration curves, yielding time‐resolved maps of optical orientation and birefringence magnitude across the entire field of view. More information about the system calibration with ITO electrodes is described in Note S3.

### 5.3 Optical Transmission Measurement

Normalized transmission was measured by removing all polarization optics in the IPOLπ setup, and the light transmitted through the sample was directly recorded by a black‐and‐white camera to measure the change of transmission intensity. In this configuration, the camera captures grayscale intensity variations corresponding to relative changes in optical transmission at each pixel. The data were processed on a per‐pixel basis in the same manner as the IPOLπ images. The measured transparency signal represents only relative temporal variations and does not provide absolute transmission values. Absolute transmission levels for the initial and poled states were independently determined using FTIR measurements averaged over the visible wavelength range and were used to calibrate the normalized transmission obtained from the camera‐based measurements. More information about the normalized transmission measurement is described in Note S5.

### 5.4 Electric Poling and Depoling

Before electric poling, the samples were heated beyond their Curie temperature to ensure the previous poling state was erased. The samples were heated at a rate of 2°C/min to 260°C, where it was maintained for 15 minutes. The cooling is controlled at 4 °C/min to room temperature. The aluminium foil was wrapped around samples to short the electrodes on opposite sides.

Electric fields were generated using a function generator (Keysight 33500B) connected to a high‐voltage amplifier (Trek 10/10B‐HS), capable of delivering both alternating (AC) and direct (DC) fields up to ±4 kV cm^−^^1^. The waveform and the camera were synchronized through a dual‐channel trigger to ensure time‐resolved correlation between optical and electrical signals. The electric field was applied along the [110] direction of the crystal through the transparent ITO electrodes. The camera recorded images at 100 frames per second. For AC poling (ACP) experiments, a sinusoidal electric field of 1 Hz and an amplitude of 5 kV cm^−^^1^ was applied. Continuous imaging captured the dynamic evolution of birefringence and domain orientation over several cycles. For DC poling (DCP), a constant electric field of 5 kV cm^−^^1^ was applied for 100 s. Electrical depoling was conducted on both AC‐ and DC‐poled samples using a low‐frequency alternating field (0.5 Hz) with an amplitude close to the coercive field (∼2.5 kV cm^−^^1^)

### 5.5 Data Processing

All colour frames were converted into hue–saturation–value (HSV) space using MATLAB. The hue component was transformed into orientation angle θ via the calibration curve, while the brightness and saturation components were used to estimate phase retardation Δ. The spatial average and histogram of θ were computed for each frame to generate time‐resolved domain‐orientation distributions. Gaussian fitting was applied to extract the main and secondary domain orientations, enabling quantitative tracking of the domain‐wall dynamics. Data analysis and figure generation were performed using MATLAB and Origin.

### 5.6 Statistical Information

Statistical analysis was performed using custom scripts implemented in MATLAB, following the same data processing and analysis procedures established in our previous IPOLπ methodology work [54]. Optical orientation distributions were obtained from pixel‐wise orientation maps and fitted using Gaussian functions. Error bars shown in the figures represent the uncertainty of the fitted peak positions, as obtained from the Gaussian fitting procedures.

The exact sample size (*n*) corresponds to the number of individual pixel points in each captured image. The images were recorded at a fixed magnification of 36× with a camera resolution of 1200 × 800 pixels (112 pixels per 100 um). Time‐resolved data points were acquired at a temporal resolution of 0.01 s per frame (100 fps). These points were not treated as independent statistical replicates.

For in‐situ poling measurements, results were recorded using a single continuous experimental sequence consisting of three successive poling–depoling cycles (such as ACP‐EDP or DCP‐EDP). This sequential cycling was used to examine the reversibility and repeatability of the domain‐wall evolution within the same sample. Additional experiments were performed on three unpolished specimens with the same dimensions and ITO‐electrode configuration, and the main ACP, DCP, and EDP trends were consistent with the results presented in the main text. Because the unpolished specimens contained additional surface‐pattern backgrounds from manufacturing, the results presented in the main text were obtained from a double‐sided, well‐polished specimen.

## Author Contributions

**Xiaoning Jiang**, **Jun Liu**, and **Kara Peters**. Conceptualization: Data curation: **Ziqi Wang**, **Anastasia Timofeeva**, **Zhengze Xu**, and **Sipan Liu**. Formal analysis: **Ziqi Wang** and **Anastasia Timofeeva**. Funding acquisition: **Xiaoning Jiang**, **Jun Liu**, **Kara Peters**, and **Eunkyoung Shim**. Investigation: **Ziqi Wang**, **Zhengze Xu**, **Sipan Liu**. Methodology: **Anastasia Timofeeva**, **Ziqi Wang**, and **Zhengze Xu**. Project administration & Supervision: **Xiaoning Jiang**, **Jun Liu**, and **Kara Peters**. Resources: **Yusen Pei**, **Franky So, Reece Henry**, and **Brendan O'Connor**. Validation: **Ziqi Wang** and **Zhengze Xu**. Writing – original draft: **Ziqi Wang**, **Anastasia Timofeeva**, and **Zhengze Xu**. Writing – review & editing: **Jun Liu**, **Xiaoning Jiang**, and **Kara Peters**.

## Funding

Z. W., X. J., and J. L. acknowledge the primary funding support from the National Science Foundation (Grant No. DMR2011978); Air Force Office of Scientific Research (Grant No. FA95502310311); A.T., H.E., E.S., and K.P. acknowledge the funding support from the Nonwovens Institute of the North Carolina State University (Project No. 21255SB); Z.X., S.L., and X.J. also acknowledge the funding support from the Office of Naval Research (Grant No. N000142112058); National Science Foundation (Grant No. DMR2309184); Y.P. and F.S. acknowledge the funding support from the Office of Naval Research (Grant No. N000142312001); B.O. and R.H. acknowledge the funding support from the Office of Naval Research (Grant No. N000142412101).

## Conflicts of Interest

The authors declare no conflicts of interest.

## Supporting information




**Supporting File 1**: advs76513‐sup‐0001‐SuppMat.docx.

**Supporting File 2**: advs76513‐sup‐0002‐VideoS1‐S6.zip.

## Data Availability

The data that support the findings of this study are available from the corresponding author upon reasonable request.
