# Supplementary material for: Real‐Time Ferroelectric Domain Wall Dynamics During Electric Poling and Depoling
Source: Adv Sci (Weinh). 2026 Jul 9:e76513. Online ahead of print. doi: 10.1002/advs.76513 (PMC13348344; doi:10.1002/advs.76513)
Supplement: Supplementary file 1 — Supporting File 1: advs76513‐sup‐0001‐SuppMat.docx. [file ADVS-9999-e76513-s002.docx]

Supporting Information

Real-time ferroelectric domain wall dynamics during electric poling and depoling

Ziqi Wang^#^, Zhengze Xu^#^, Anastasia Timofeeva^#^, Hossam Elnaggar, Sipan Liu, Yusen Pei, Reece Henry, Brendan O'Connor, Eunkyoung Shim, Franky So, Kara Peters*, Xiaoning Jiang*, Jun Liu*

Z. Wang, Z. Xu, A. Timofeeva, H. Elnaggar, S. Liu, R. Henry, B. O’Connor, K. Peters, X. Jiang, J. Liu

Department of Mechanical and Aerospace Engineering, North Carolina State University, Raleigh, NC 27695, USA
E-mail: kjpeters@ncsu.edu; xjiang5@ncsu.edu; jliu38@ncsu.edu

Y. Pei, F. So
Department of Materials Science and Engineering, North Carolina State University, Raleigh, NC 27695, USA

E. Shim
Department of Textile Engineering, North Carolina State University, Raleigh, NC 27695, USA

[#] These authors contributed equally to this work.

Supplementary Note 1. Overview of the IPOLπ method

The instant polarized light microscopy (IPOLπ) technique used in this study follows the same optical principle and calibration procedure described in our previous work^[1–7]^. A 6500 K white LED is first converted into a nearly circularly polarized beam using a linear polarizer and a quarter-wave plate (QWP) fixed at 45°. After passing through the birefringent specimen, the transmitted light enters a z-cut quartz rotator (total thickness 6 mm in this work), which introduces a wavelength-dependent optical rotation^[8]^. This rotation spectrally separates different polarization components across the visible range. The beam then passes through a horizontal analyzer before being recorded by a color camera (Phantom v1612). The spectra signal captured by the camera is processed through the separate RGB sensors, where RGB (Red, Green, Blue) denotes the additive primary colors in digital imaging, and it will produce a specific color output dependent on the polarization properties of the imaged sample (**Fig. S1**). This configuration ensures that the change of experimentally recorded RGB response from camera images mainly depends on the local optical orientation ($\alpha_{s}$) and phase retardation ($\phi_{s}$) of the specimen.

For image processing, the recorded RGB frames were transformed into the HSV (hue-saturation-value) space, which provides a convenient representation to visually enhance spatial variations in the optical response and, more importantly, to facilitate quantitative separation of orientation-related and retardation-related information^[1]^. The hue channel is primarily governed by the sample alignment and was used to extract the local optical orientation ($\alpha_{s}$). The value channel reflects pixel brightness and depends on both alignment and retardation. While IPOLπ does not yield an absolute $\phi_{s}$, the value channel exhibits a sinusoidal dependence on $\phi_{s}$ (maximum at 90°) and was therefore used as a retardation-related proxy $sin(\phi_{s})$ to track birefringence changes during poling.

The electric poling function was performed using a custom-designed fixture (**Fig. S2**), in which the copper tapes were placed on both sides of the sample (over the ITO-coated surfaces) to serve as electrical contacts. Power cables were clamped onto the copper tapes and secured with electrical tape to ensure insulation and maintain a stable mechanical fixture and reliable electrical connection throughout the measurements.


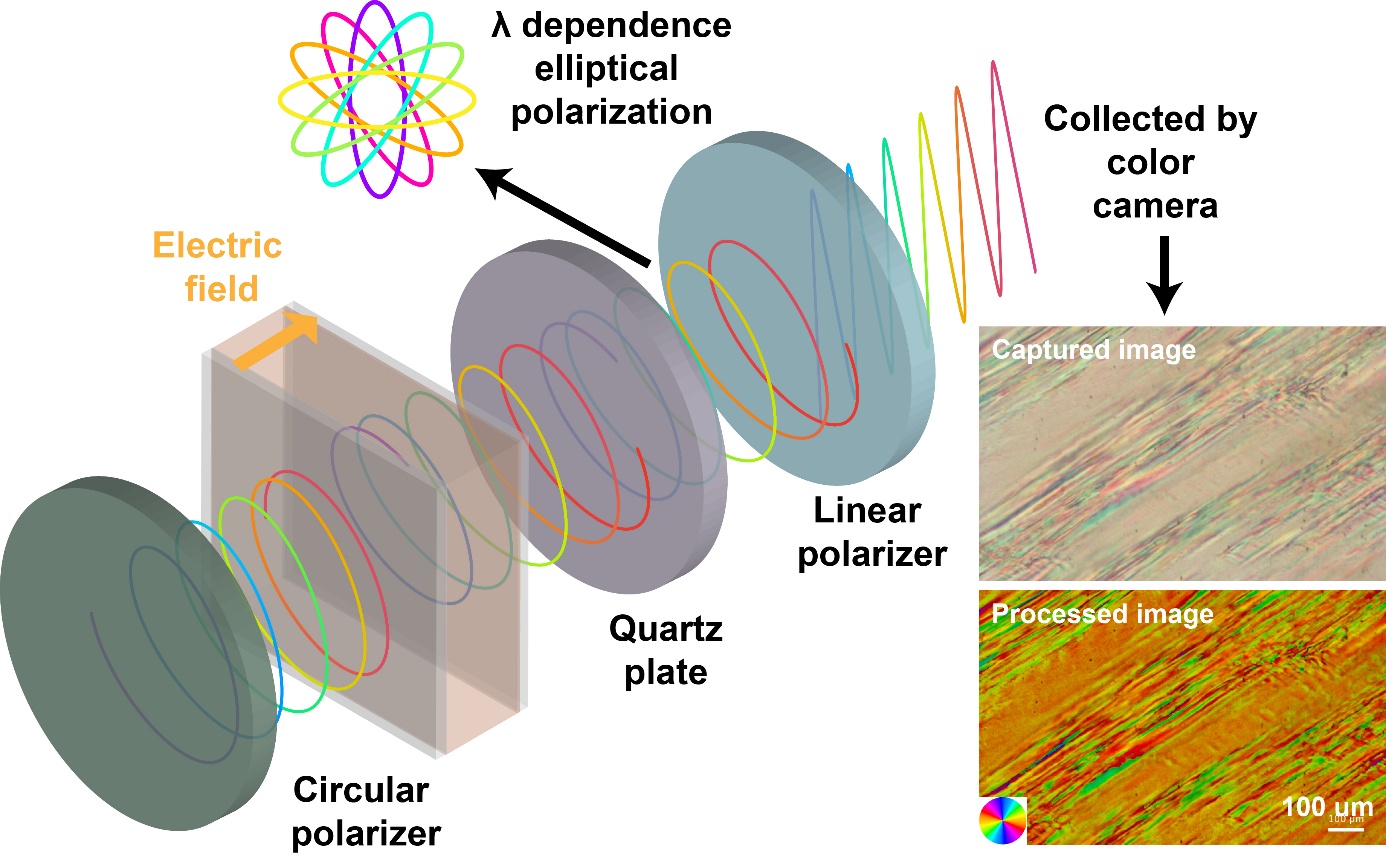


**Figure S1.** Schematic of the IPOLπ optical train and captured images. The optical observation direction is parallel to the applied electric field. The recorded color image is processed using a calibrated angle–colour relationship to extract the in-plane optical orientation. The image brightness is used to derive the phase-retardation-related signal $\sin(\phi_{S})$.


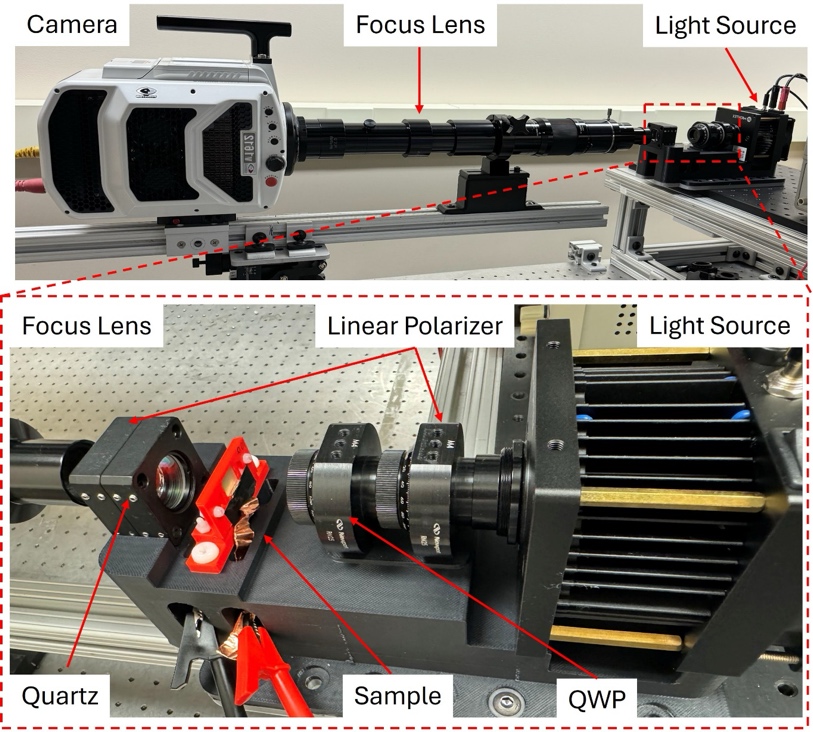


**Figure S2.** Photograph of the in-situ IPOLπ poling setup at North Carolina State University. Representative photos showing the optical components and the electrical poling fixture while continuously recording IPOLπ images.

Supplementary Note 2. Experimental validation of the IPOLπ poling fixture

Each sample was first treated by thermal annealing before electric poling. A thermally annealed specimen was first characterized and regarded as the initial state and then subjected to the same electrical loading protocols used in the in-situ studies. After the thermal annealing, the sample was loaded with the ACP, DCP, or EDP electrical waveforms. ACP and DCP were applied with the amplitudes and frequencies listed in **Table S1**. For the EDP case, a unipolar triangle wave, opposite to the polarization (positive piezoelectricity) direction, was applied to remove the domains previously formed. The ACP and DCP voltage has been previously optimized to achieve effective poling^[9]^. The EDP voltage was chosen based on prior measurements showing significant reduction in piezoelectricity^[10]^. Because the poling was conducted in air, a lower electric-field density was selected to prevent sample damage. Similarly, the DCP ramp rate was kept fixed in the present in-situ measurements. Both higher electric-field density and faster ramping to the target DC field followed by sustained field holding increased the risk of sample fracture under this air-based optical poling configuration.

To ensure that the custom in-situ IPOLπ poling fixture provides stable electrical contact and yields representative poling outcomes, we conducted benchmark tests prior to the in-situ imaging experiments. The samples’ dielectric and piezoelectric properties were measured using both the commercial poling system (aixACCT) and the IPOLπ fixture setup to compare. The [110] sample tested has a dimension of 12 mm × 12 mm × 0.5 mm. ITO is deposited on both sides of the [110] direction. These results shown in **Table S2** were measured directly after poling without aging, because the objective of this study is to observe the in-situ movement in domain wall motion rather than stable piezoelectric material properties. The results show that the custom poling fixture achieves similar poling effect.

**Table S1.** Parameters of the ACP, DCP, and EDP electrical field signals applied to the sample

| Parameter | ACP Value | DCP Value | EDP Value |
| --- | --- | --- | --- |
| Frequency | 1 Hz | / | 0.05 Hz |
| Electric field (peak-to-peak) | 10 kV/cm | 5 kV/cm | 5.5 kV/cm |
| Voltage (peak-to-peak) | 340 V | 170 V | 187 V |
| Cycles | 20 | / | 1 |

**Table S2.** Dielectric constant ($\varepsilon_{33}^{T}/\varepsilon_{0}$) and d_33_ (pC/N) comparison between different poling setups

| Poling Method | Equipment | *d_33_* (pC/N) | Dielectric constant ($\varepsilon_{33}^{T}/\varepsilon_{0}$) |
| --- | --- | --- | --- |
| Thermal annealed | - | 7 | 2560 |
| ACP | aixACCT | 1010 | 2280 |
|  | IPOL$\pi$ fixture | 990 | 2210 |
| DCP | aixACCT | 730 | 2940 |
|  | IPOL$\pi$ fixture | 740 | 3030 |
| EDP | aixACCT | 31 | 1880 |
|  | IPOL$\pi$ fixture | 52 | 2040 |

Supplementary Note 3. Impact of ITO transmission and the corresponding calibration for the IPOLπ system

Because IPOLπ relies on spectral encoding of polarization information, wavelength-dependent transmission variations from transparent electrodes can bias the recorded RGB (Red, Green, Blue) response if not properly accounted for. In particular, the presence of ITO electrodes introduces wavelength-dependent transmission variations that can affect the colour response by reshaping the spectrum of light reaching the camera sensor. The original hue-orientation color wheel calibration had a systematic shift when ITO electrodes are present, which would further affect the extracted optical orientation.

To isolate the specimen birefringence response from electrode-induced spectral effects, we perform an extra calibration under the same optical configuration, where two 200 nm-thick-ITO-coated glass substrates are placed on both sides of the polycarbonate reference sample. This calibration follows the previously reported polycarbonate-based hue–orientation calibration procedure^[1,2]^. **Figure S3a** compares the measured transmission spectra for bare glass and an ITO/glass/ITO sample, confirming that ITO introduces a wavelength-dependent transmission change that can affect the IPOLπ system (most noticeably at shorter wavelengths). This spectral reshaping leads to a small but measurable 5º shift in the hue–orientation mapping (**Figure. S3b**) and in the corresponding colour wheel (**Figures. S3c–d**). Here, the hue is computed from the recorded RGB values by converting the images into the HSV colour space and is used as the scalar colour metric for establishing the hue–orientation calibration. In this work, all IPOLπ analyses use the new calibrated color wheel to ensure that the extracted orientation reflects the specimen birefringence response.


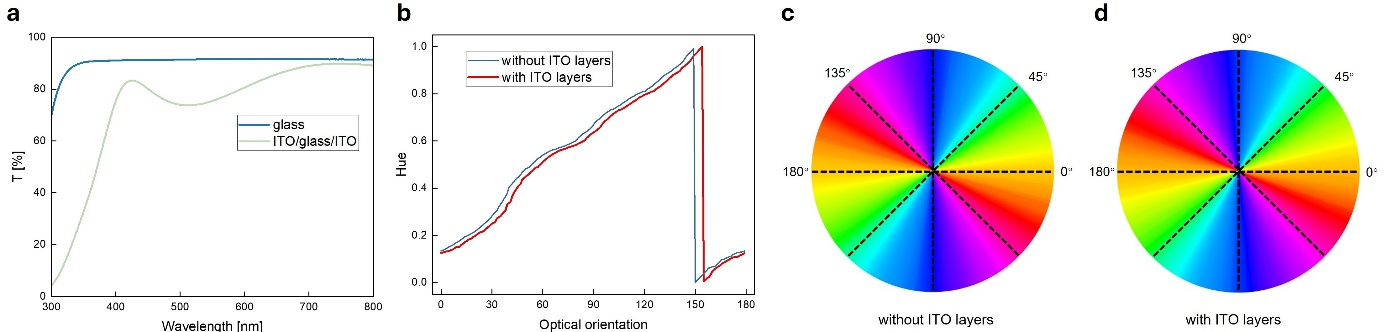


**Figure S3.** a) Measured transmission T spectra for bare glass and the ITO/glass/ITO sample, showing the wavelength-dependent attenuation introduced by ITO. b) Calibrated hue as a function of optical orientation without and with ITO layers in the same IPOLπ system. c–d) corresponding colour-wheels derived from the calibration curves without (c) and with (d) ITO layers, showing the ITO-induced shift in the effective colour response.

Supplementary Note 4. Time-resolved evolution of the phase-retardation-related signals

During ACP, the temporal evolution of both the dominant optical orientation and the phase-retardation-related signal $sin(\phi_{s})$ was extracted at each time point, as shown in **Figure. S4**. Both dominant values are obtained from Gaussian fitting histograms and represent the thickness-integrated, in-plane birefringent response. Both quantities exhibit reproducible, cycle-dependent modulations that are synchronized with the applied AC electric field. Enlarged views highlight that the most pronounced changes occur during the rising period of the electric field within each AC cycle. The sharp transitions in $sin(\phi_{s})$ are attributed primarily to field-driven reconfiguration of the thickness-integrated birefringent response associated with domain-wall motion and rearrangement along the electric-field direction.


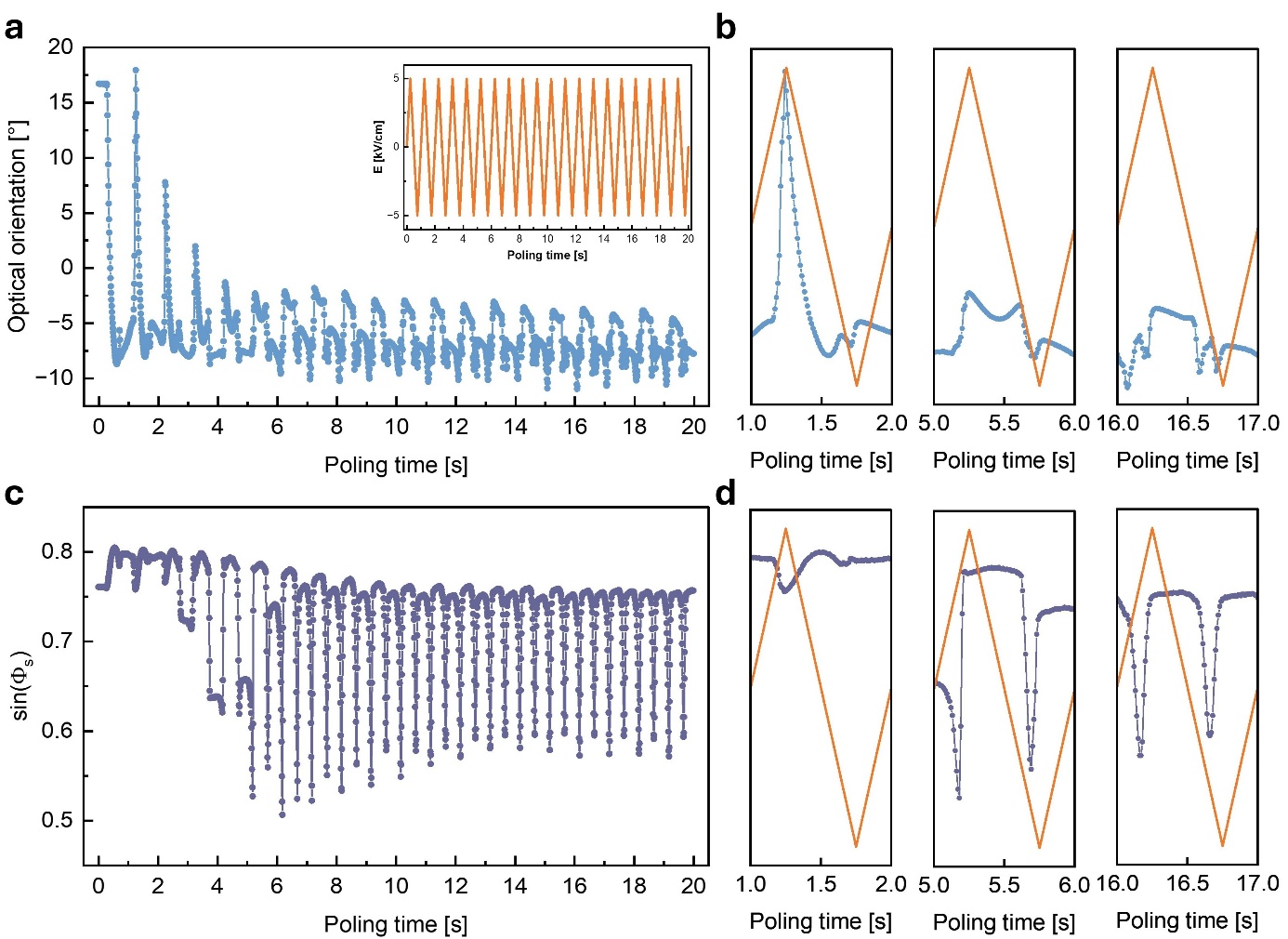


**Figure S4.** Time-resolved dominant optical orientation and phase-retardation-related signal ($\sin(\phi_{S})$) during entire ACP process. a) Time-resolved evolution of the dominant optical orientation during the AC poling, extracted from Gaussian fitting to the orientation histograms. The inset shows the corresponding AC poling electric field applied to the sample. b) Enlarged views of the optical orientation evolution under different AC poling cycles. c) Time-resolved evolution of the dominant phase-retardation-related signal ($\sin(\phi_{S})$) during the AC poling, extracted from Gaussian fitting to the orientation histograms. d) Enlarged views of the $\sin(\phi_{S})$ evolution under different AC poling cycles.

Supplementary Note 5. Normalized transmission measurement

To verify that the observed colour and brightness evolution under poling is dominated by polarization/birefringence changes rather than gross intensity drift, we quantify normalized transmission by removing polarization-selective optics and measuring the camera intensity response under otherwise identical imaging conditions. **Figure S5** summarizes the normalized transmission results.


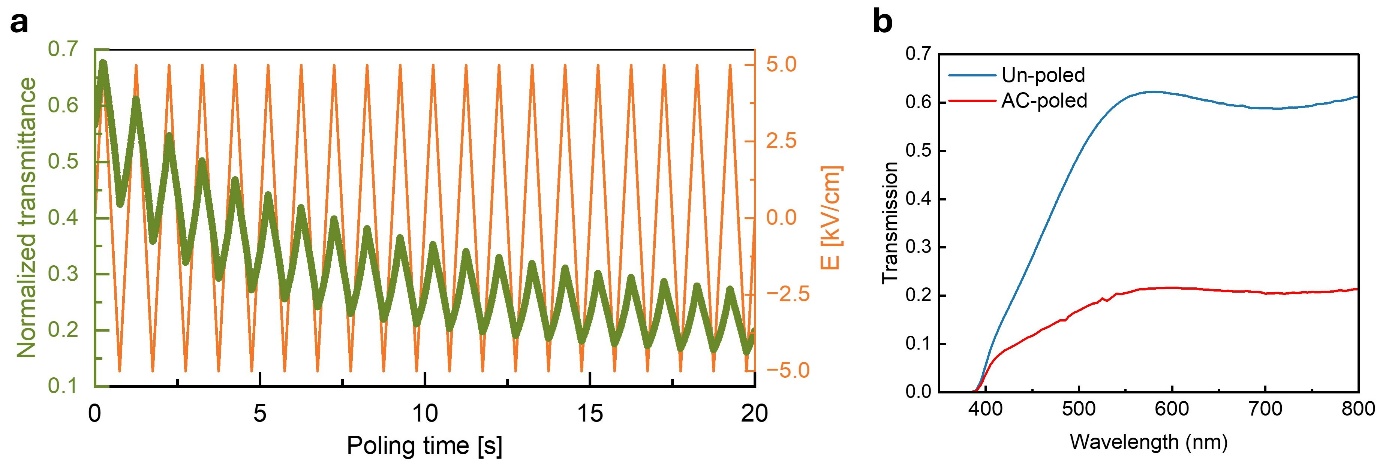


**Figure S5.** Normalized transmission measurement during entire ACP process. a) Time-resolved transmission signal recorded during ACP, plotted together with the applied AC electric field. b) Representative transmission spectra acquired at before ACP state and after ACP state.

Supplementary Note 6. In-situ IPOLπ images captured during ACP

Representative IPOLπ image acquired during alternating-current poling (ACP) are provided in **Figures. S6** and **S7**. **Figure S6** summarizes the domain-wall evolution across the full ACP process, with selected frames including the initial response, the progressive cycle-dependent reconfiguration, and the subsequent cycle-stabilized state; the time markers in **Figure. S6a** correspond to the image frames in **Figures. S6 b–f**. **Figure S7** further resolves the within-cycle dynamics by showing representative images from a single AC cycle at defined phases of the waveform (**Figure. S7a**), highlighting that the most pronounced colour/contrast changes and domain-wall rearrangements occur predominantly during the rising branches following the zero crossings, whereas comparatively reduced changes are observed near the extrema. Together, these ACP image sequences provide a visual complement to the time-resolved optical metrics and histogram-based analyses reported in the main text by directly illustrating the synchronized, field-driven evolution of domain-wall textures under ACP. After the ACP process, the main ACP-induced domain wall features remained visible and stable.


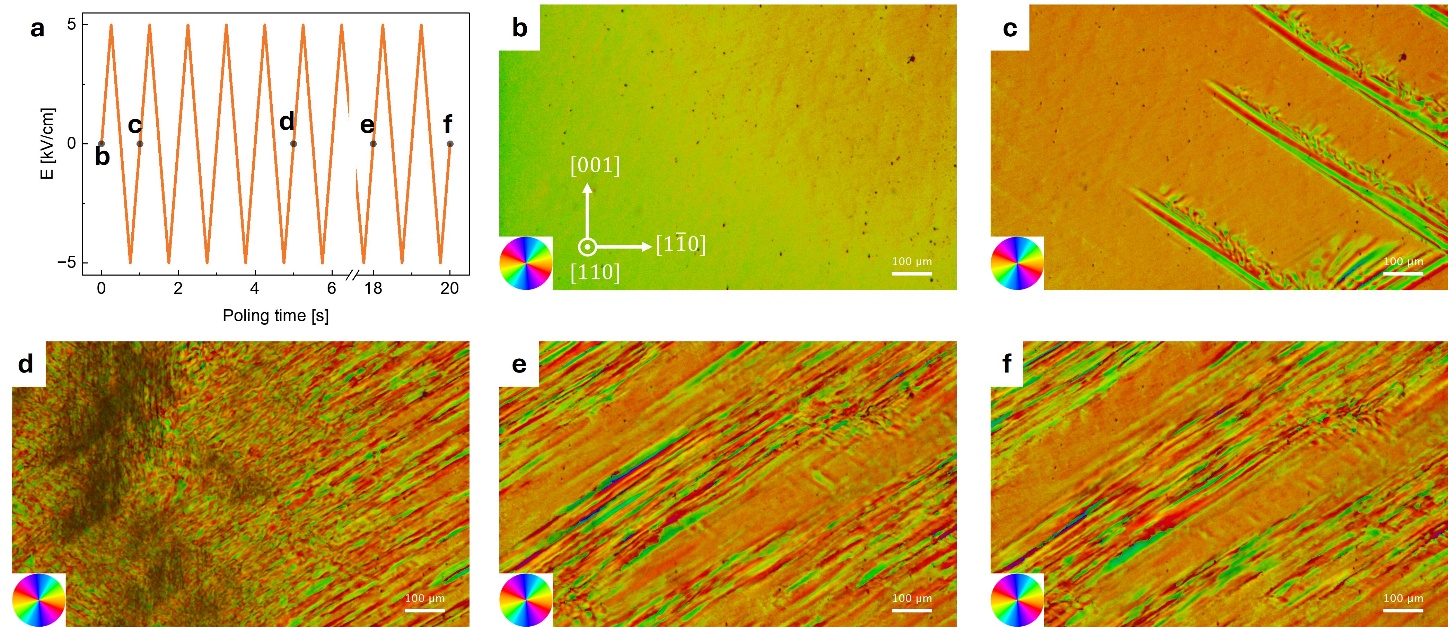


**Figure S6.** Representative IPOLπ images during ACP. a) Applied ACP field with markers indicating the time points of the captured images in (b–f). b–f) Selected IPOLπ images illustrating the evolution of domain-wall patterns from the initial response to the stabilized ACP configuration.


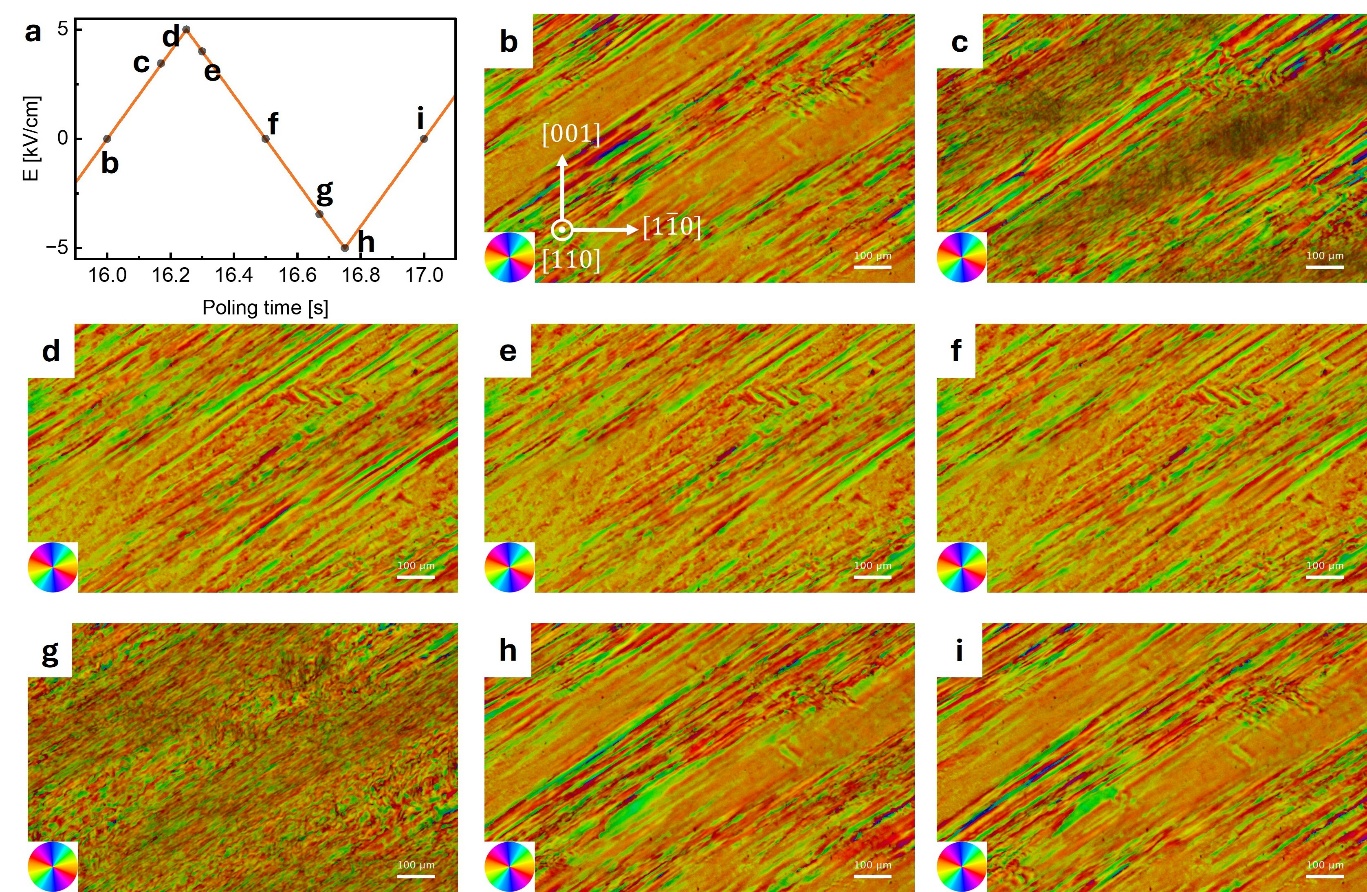


**Figure S7.** Representative single-cycle IPOLπ images in ACP. a) Representative single-cycle ACP field during ACP process with markers indicating the time points of the captured images in (b–i). b–i) Selected IPOLπ images showing within-cycle evolution synchronized to the applied field, highlighting rapid field-driven changes near the rising branches and comparatively reduced changes near the extrema.

Supplementary Note 7. In-situ IPOLπ images captured during DCP

Representative IPOLπ image captured during direct-current poling (DCP) are provided in **Figure. S8**. The displayed frames were selected to capture the ramp-up, peak-hold, and ramp-down periods of the unidirectional DC electric field, allowing the domain-wall textures to be compared across distinct electrical stages. Consistent with the time-resolved optical metrics, the most pronounced colour/contrast changes occur during the initial field-increasing period, while the images become comparatively uniform during the constant-field hold, indicating that the domain configuration rapidly stabilizes under a steady DC field. **Figure S9** presents a polarity-reversal control (opposite DC polarity with identical magnitude and timing), which reproduces the transient response with the expected inversion, supporting that the observed behaviour reflects intrinsic field-driven reconfiguration rather than imaging artefacts.


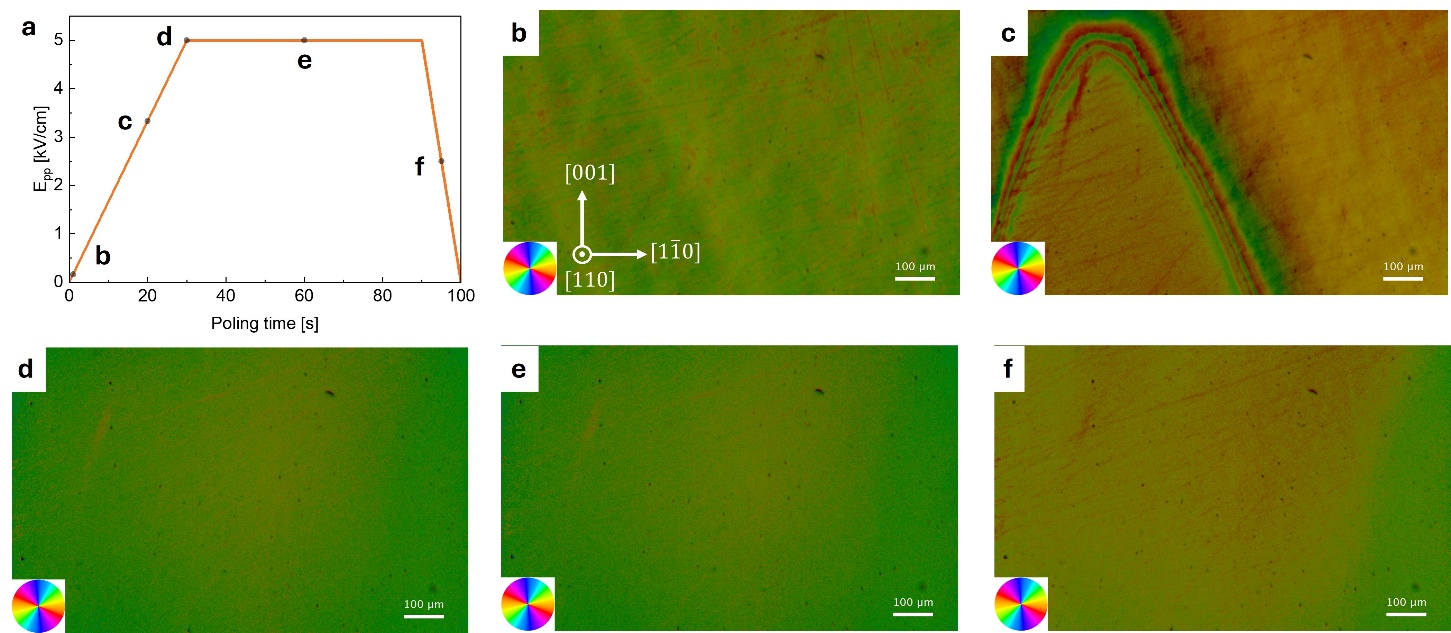


**Figure S8.** Representative IPOLπ images during DCP. a) Applied DCP field with markers indicating the time points of the captured images in (b–f). b–f) Selected IPOLπ images including ramp-up, peak/hold, and ramp-down. The images show that the dominant optical changes occur primarily during the field increasing part, while the response becomes comparatively steady during the constant-field hold.


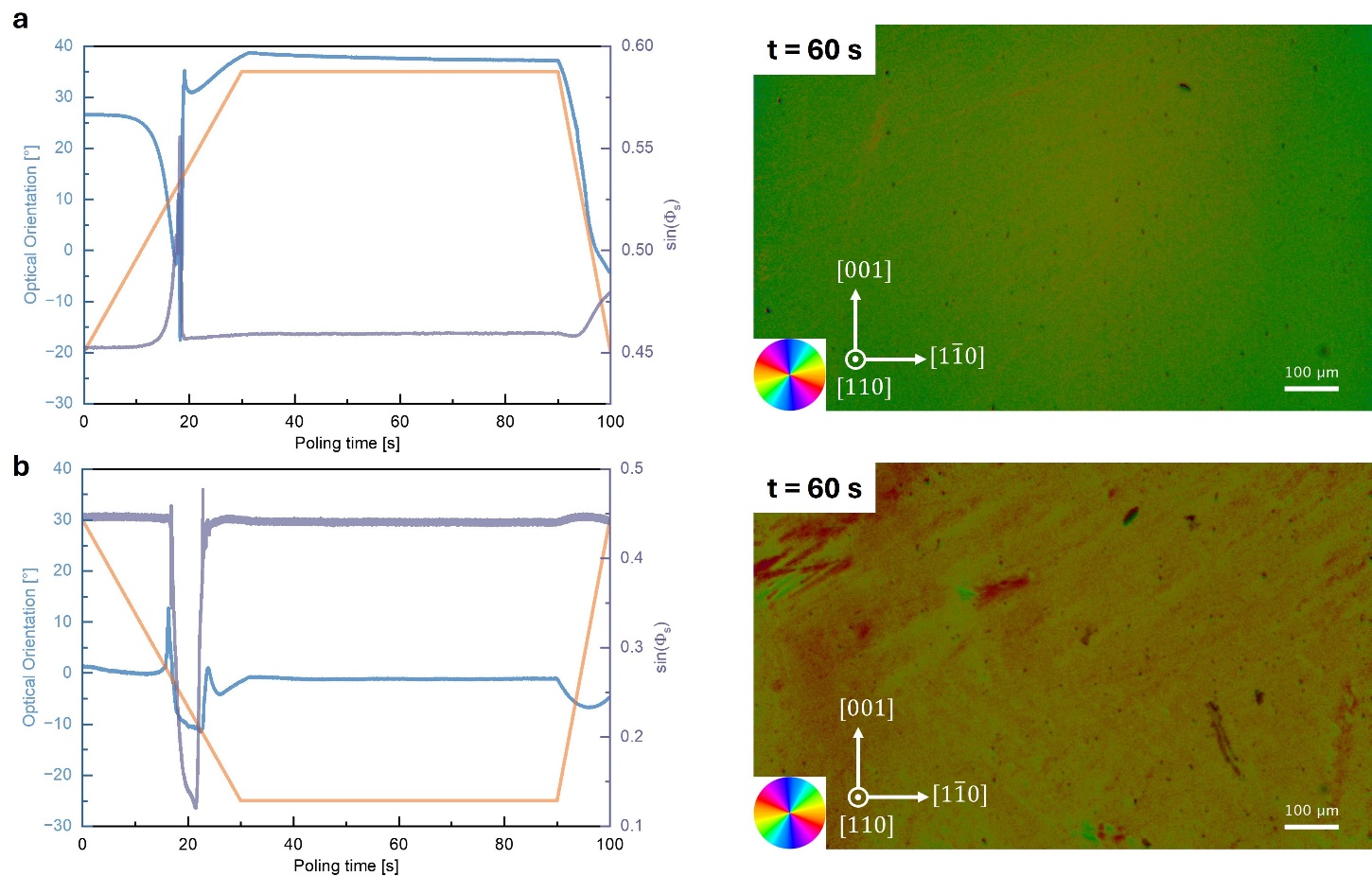


**Figure S9.** DCP response under opposite electric-field directions. Time-resolved optical response and representative images acquired under DCP protocols with opposite electric-field polarities. The polarity-dependent transient response supports that the observed behavior reflects intrinsic, field-driven reconfiguration dynamics rather than imaging artifacts.

Supplementary Note 8. In-situ IPOLπ images captured during EDP

Representative IPOLπ images captured during electrical depoling (EDP) are shown in **Figure. S10** for different initial poling histories (after ACP versus after DCP). In both cases, the dominant image-level changes are concentrated in the field-increasing portion of the depoling waveform, consistent with a field-activated relaxation process. After the completion of EDP, the images become optically more homogeneous, and the prominent domain-wall traces formed during prior poling are strongly suppressed. Notably, residual mesoscale textures remain dependent on the initial poled state, indicating that EDP can drive convergence of the global optical-orientation statistics while preserving history-dependent domain-wall topology.


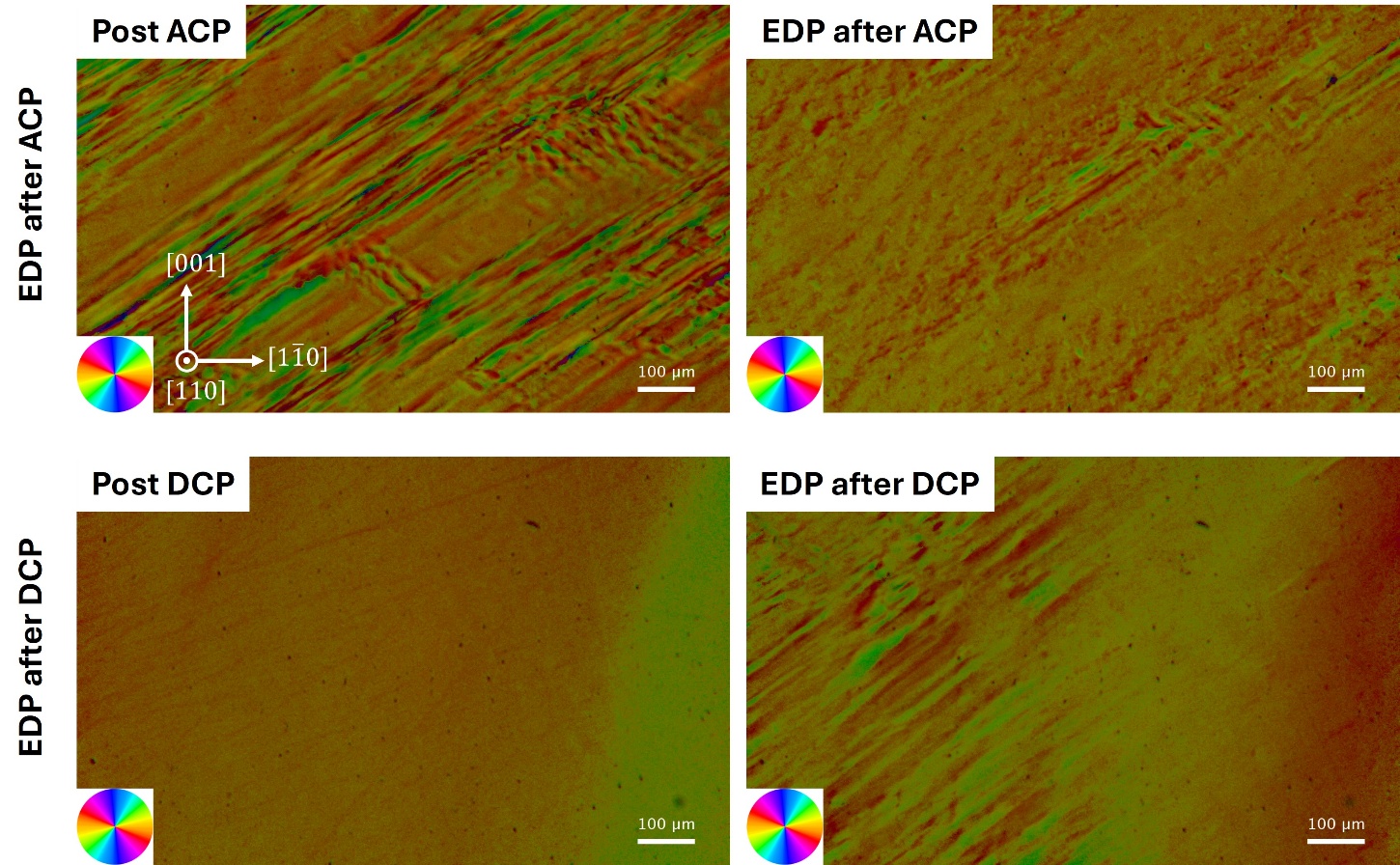


**Figure S10.** IPOLπ images during electrical depoling (EDP) after different initial poling histories. Selected IPOLπ images acquired during EDP. Panels compare outcomes starting from different initial poling histories (after ACP vs after DCP). The relaxation pathways and domain-wall features depend on the initial poling history.

Supplementary Note 9. DCP following EDP with the same field direction

To assess whether the post-EDP configuration retains a significant driving force for further unidirectional realignment, we applied a subsequent DCP using the same field direction as that employed during the EDP cycle and compared its response with that obtained under reversed-polarity post-EDP DCP. If strong residual nonequilibrium polarization remained after EDP, a renewed unidirectional bias would be expected to induce a rapid reconfiguration of domains, shown in the evolution of the extracted optical orientation together with a correlated change in $\sin\left( \phi_{S} \right)$.

As shown in **Figure. S11a**, the optical orientation continues to evolve gradually during the DCP process, while the change shows a delay in poling time and the characteristic abrupt transition near 20 s is no longer evident. The $\sin\left( \phi_{S} \right)$ also shows a stable feature without the transient features associated with the field ramping stages. The domain-wall patterns induced by the preceding EDP process progressively weaken and disappear. This result indicates a field-assisted reorganization of the residual domain structure. The processed IPOLπ images show that the ACP-like domain wall patterns generated during the preceding EDP process progressively weaken and disappear under the same-polarity DCP, indicating a field-assisted reorganization and homogenization of the residual domain structure rather than a fresh, large-scale switching event.

These results suggest that the subsequent same-polarity DCP primarily promotes slow relaxation and annealing of the EDP-induced domain configuration, instead of driving strong unidirectional realignment. The post-EDP state therefore corresponds to a near-relaxed configuration, in which only weak residual rearrangements persist under the same field direction.


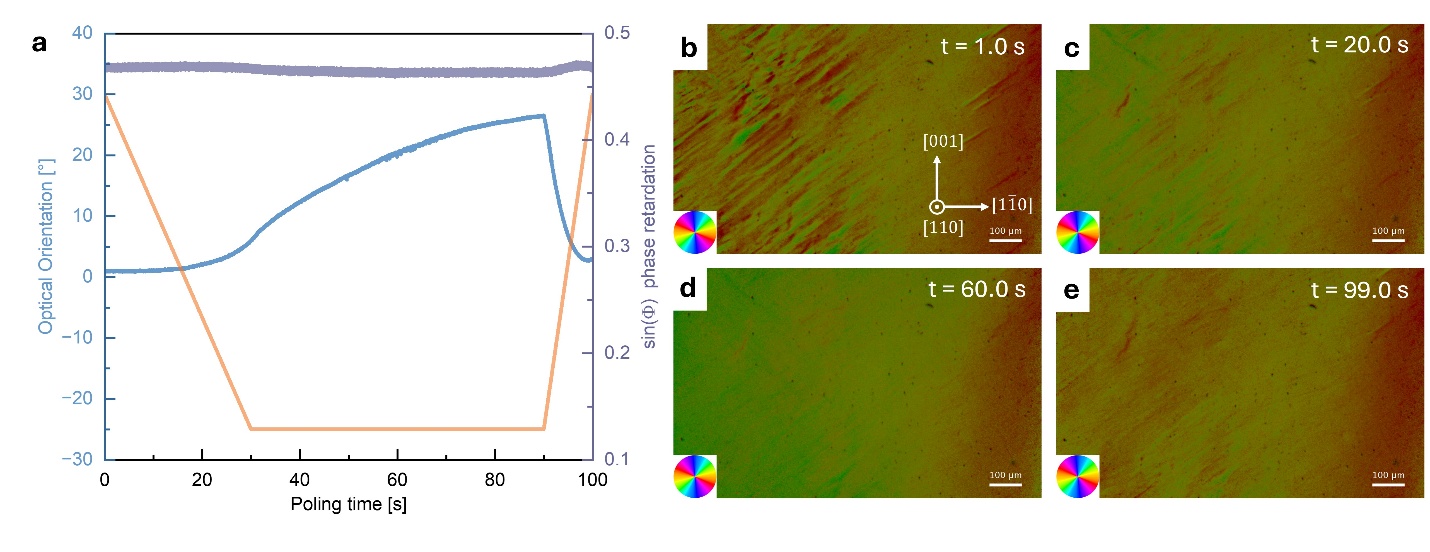


**Figure S11.** Response to a subsequent same-polarity DCP applied after an EDP cycle. a, Time evolution of the extracted optical orientation and $\sin\left( \phi_{S} \right)$ during the post-EDP DCP (same field direction) process. The optical orientation exhibits a delayed onset and a slow, continuous evolution, without the abrupt transition near 20 s, and $\sin\left( \phi_{S} \right)$ remains stable during the whole process. b–e, Representative IPOLπ images at selected stages during the same-polarity post-EDP DCP process. The ACP-like domain wall patterns generated during the preceding EDP step progressively weaken and disappear during this DCP process, indicating a field-assisted reorganization and homogenization of the residual domain structure rather than a fresh large-scale switching event.

Supplementary Note 10. DCP-induced domain dynamics starting from different initial states (thermally depoled *vs* electrically depoled)

To distinguish the post-EDP state from conventional thermal depoling, we performed DCP after thermal depoling and EDP and compared the resulting optical-orientation maps at matched times, as summarized in **Fig. S12**. While both conditions exhibit their primary reconfiguration during the DC ramp-up, the mesoscale textures differ: the post-EDP specimen can transiently access ACP-like ~35° domain-wall traces during the field-increasing stage, whereas the thermally depoled specimen shows a more uniformly evolving response. These comparisons indicate that EDP does not necessarily reproduce the same relaxed domain configuration as thermal depoling, even when the subsequent unidirectional poling protocol is identical.


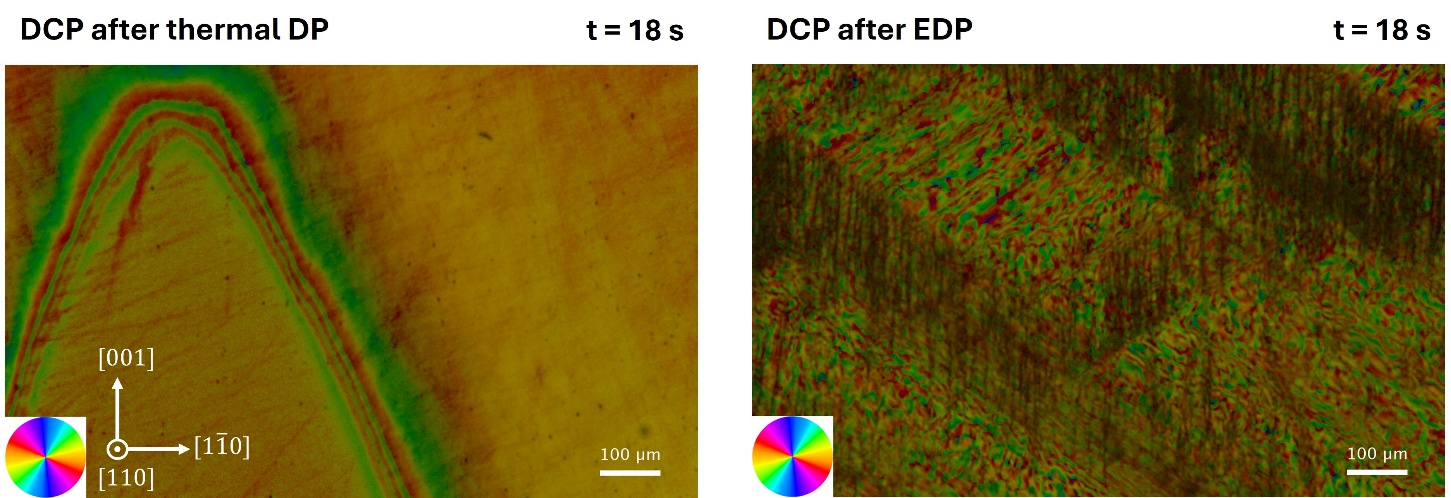


**Figure S12.** Comparison of DCP response after thermal depoling versus after EDP. Optical orientation images acquired at matched times during DCP from thermal de-poled state (left) and EDP state (right). Differences in the resulting mesoscale textures indicate that EDP does not necessarily reproduce the same domain configuration as thermal depoling.

Supplementary Note 11. Repeatability assessment under repeated ACP–EDP and DCP–EDP cycling

Repeatability under repeated electrical histories was evaluated using alternating poling–depoling sequences recorded continuously on the same specimen. Specifically, ACP and EDP were alternated for multiple rounds (ACP–EDP–ACP–EDP**–**ACP**–**EDP), and an analogous DCP–EDP cycling protocol was performed under the same imaging and analysis conditions. **Figures S13** and **S14** summarize representative cycles, showing that both the extracted optical orientation and phase-retardation-related signals are reproducible from cycle to cycle once the protocol is fixed. These multi-cycle tests confirm that the reported responses are not isolated events, but rather repeatable field-programmed reconfiguration pathways.


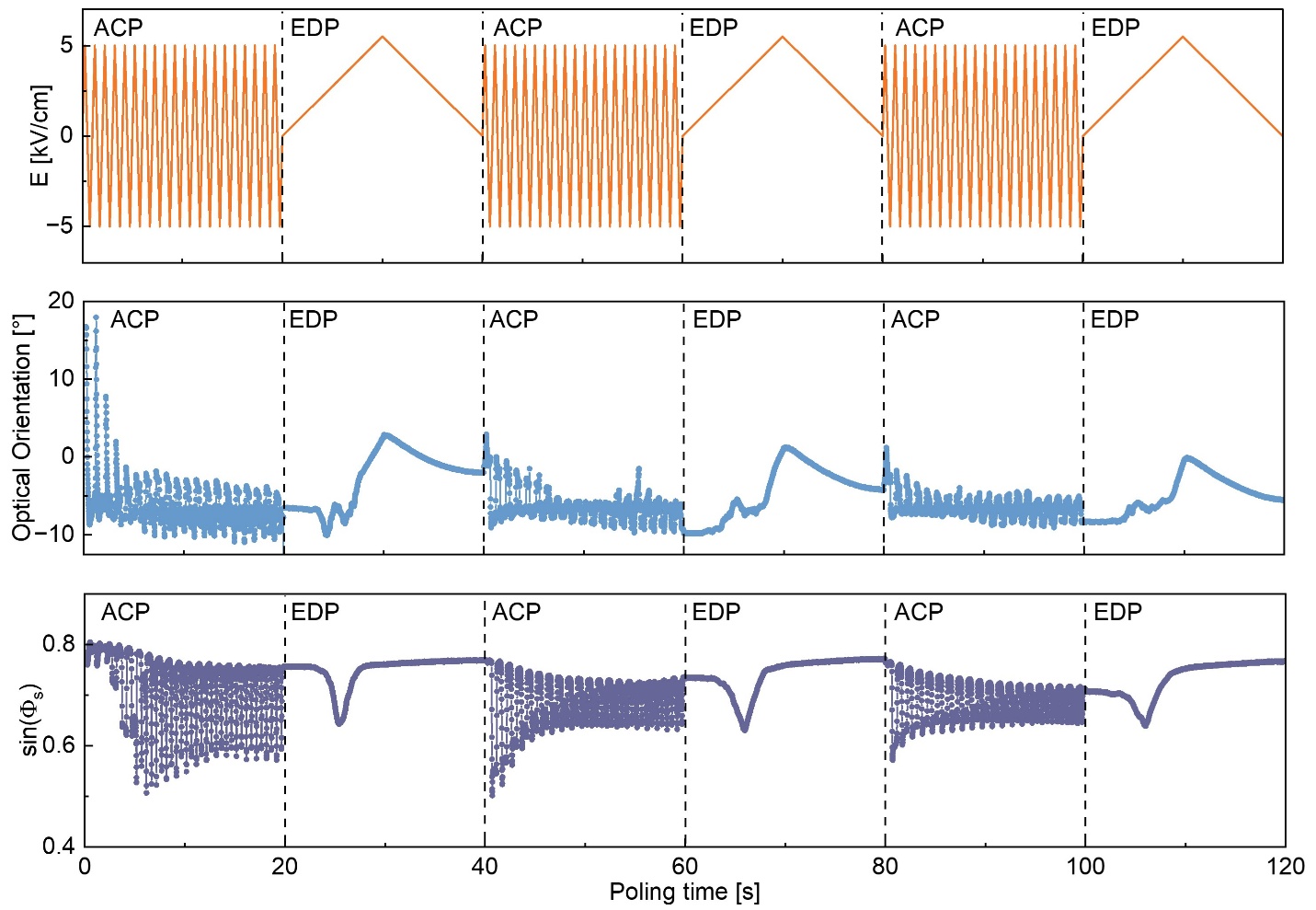


**Figure S13.** Repeatability assessment under alternating ACP–EDP cycling.


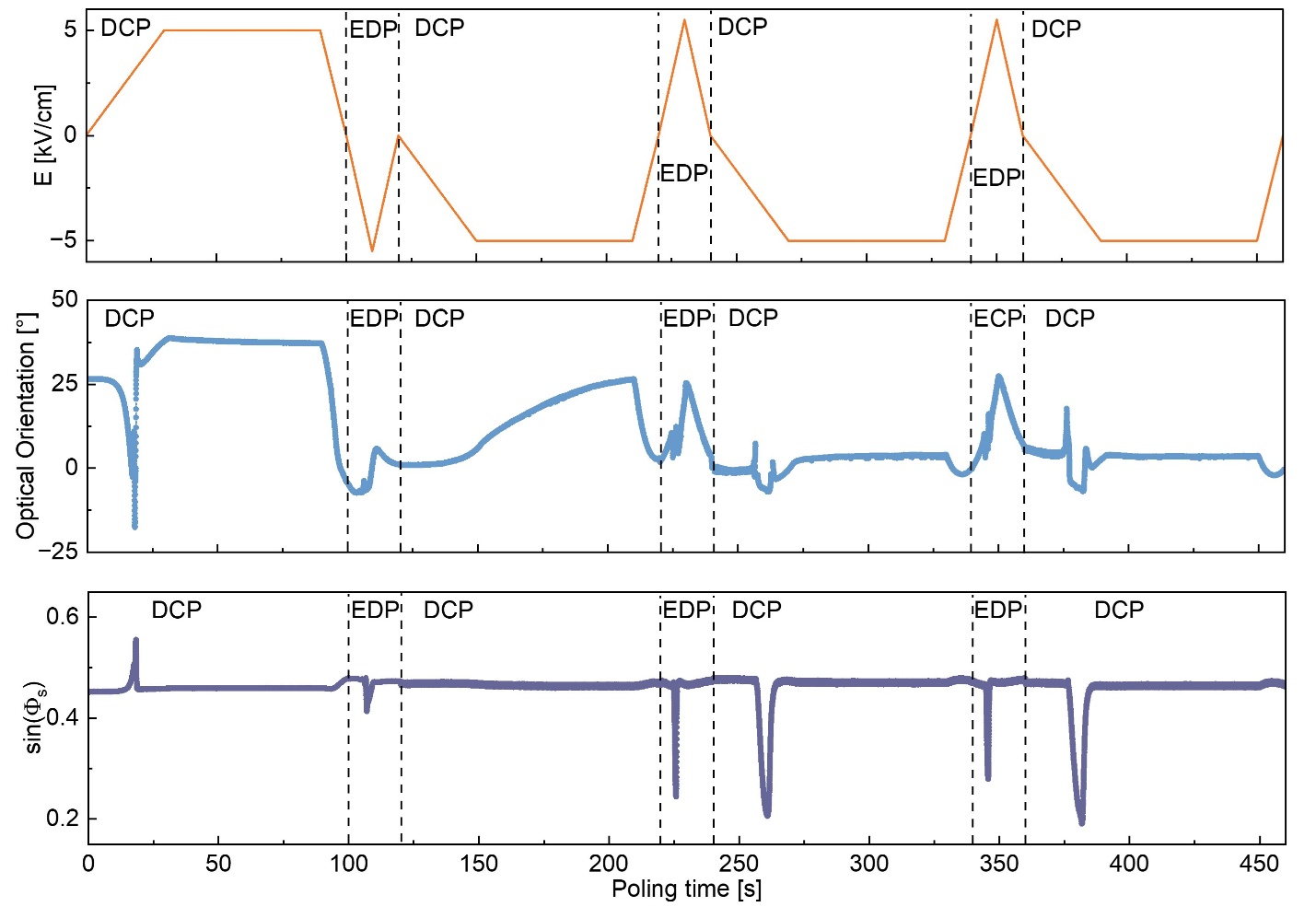


**Figure S14.** Repeatability assessment under alternating DCP–EDP cycling.

**References**

1. A. Timofeeva, Z. Wang, Z. Xu, et al., “*In Situ* Imaging of Domain Walls in Ferroelectric Single Crystals by Instant Polarized Light Microscopy,” *Review of Scientific Instruments* *97*, no. 3 (2026): 033701.

2. P. Y. Lee, H. Schilpp, N. Naylor, S. C. Watkins, B. Yang, and I. A. Sigal, “Instant Polarized Light Microscopy Pi (IPOLπ) for Quantitative Imaging of Collagen Architecture and Dynamics in Ocular Tissues,” *Optics and Lasers in Engineering* *166* (2023).

3. B. Yang, P. Y. Lee, Y. Hua, et al., “Instant Polarized Light Microscopy for Imaging Collagen Microarchitecture and Dynamics,” *Journal of Biophotonics* *14* (2021).

4. M. Shribak, “Polychromatic Polarization Microscope: Bringing Colors to a Colorless World,” *Scientific Reports* *5* (2015).

5. N. J. Jan, K. Lathrop, and I. A. Sigal, “Collagen Architecture of the Posterior Pole: High-Resolution Wide Field of View Visualization and Analysis Using Polarized Light Microscopy,” *Investigative Ophthalmology & Visual Science 58* (2017): 735–744.

6. N.-J. Jan, J. L. Grimm, H. Tran, et al., “Polarization Microscopy for Characterizing Fiber Orientation of Ocular Tissues,” *Biomedical Optics Express* *6* (2015): 4705.

7. M. Shribak, “Complete Polarization State Generator with One Variable Retarder and Its Application for Fast and Sensitive Measuring of Two-Dimensional Birefringence Distribution,” *Journal of the Optical Society of America A* *28*, no. 3 (2011): 410–419.

8. C. S. Hurlbut Jr., and J. L. Rosenfeld, “Monochromator Utilizing the Rotary Power of Quartz*,” *American Mineralogist* *37*, nos. 3–4 (1952): 158–165.

9. Y. Yamashita, T. Karaki, H. Y. Lee, H. Wan, H. P. Kim, and X. Jiang, “A Review of Lead Perovskite Piezoelectric Single Crystals and Their Medical Transducers Application,” *IEEE Transactions on Ultrasonics, Ferroelectrics, and Frequency Control* *69* (2022): 3048–3056.

10. H. P. Kim, M. H. Zhang, B. Wang, et al., “Electrical De-Poling and Re-Poling of Relaxor-PbTiO_3_ Piezoelectric Single Crystals without Heat Treatment,” *Nature Communications* *15* (2024).
